# Stimuli-Responsive Self-Healing Ionic Gels: A Promising Approach for Dermal and Tissue Engineering Applications

**DOI:** 10.1021/acsbiomaterials.4c02264

**Published:** 2025-02-25

**Authors:** Deepanjan Datta, Viola Colaco, Sony Priyanka Bandi, Namdev Dhas, Leela Sai Lokesh Janardhanam, Sudarshan Singh, Lalitkumar K. Vora

**Affiliations:** † Department of Pharmaceutics, Manipal College of Pharmaceutical Sciences, Manipal Academy of Higher Education, Manipal 576104, Karnataka, India; ‡ Department of Pharmacy, Birla Institute of Technology and Science (BITS) Pilani, Hyderabad Campus, Hyderabad 500078, Telangana, India; § Department of Pharmaceutical Sciences and Experimental Therapeutics, College of Pharmacy, University of Iowa, Iowa City, Iowa 52242, United States; ∥ Faculty of Pharmacy, Chiang Mai University, Chiang Mai 50200, Thailand; ⊥ Office of Research Administrations, Chiang Mai University, Chiang Mai 50200, Thailand; # School of Pharmacy, Queen’s University Belfast, 97 Lisburn Road, Belfast BT9 7BL, U.K.

**Keywords:** stimuli-responsive, self-healing, ionic interaction, ionic liquids, hydrogels, dermal, tissue engineering

## Abstract

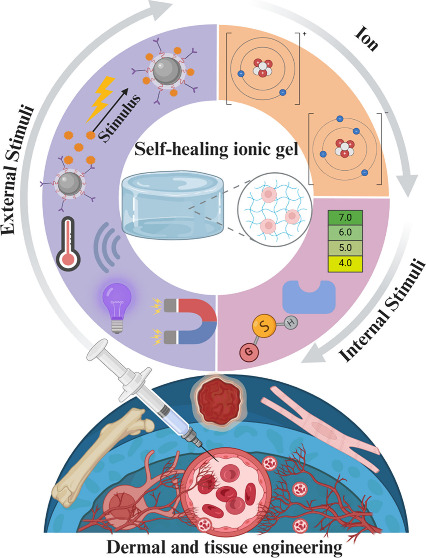

The rapid increase in the number of stimuli-responsive polymers, also known as smart polymers, has significantly advanced their applications in various fields. These polymers can respond to multiple stimuli, such as temperature, pH, solvent, ionic strength, light, and electrical and magnetic fields, making them highly valuable in both the academic and industrial sectors. Recent studies have focused on developing hydrogels with self-healing properties that can autonomously recover their structural integrity and mechanical properties after damage. These hydrogels, formed through dynamic covalent reactions, exhibit superior biocompatibility, mechanical strength, and responsiveness to stimuli, particularly pH changes. However, conventional hydrogels are limited by their weak and brittle nature. To address this, ionizable moieties within polyelectrolytes can be tuned to create ionically cross-linked hydrogels, leveraging natural polymers such as alginate, chitosan, hyaluronic acid, and cellulose. The integration of ionic liquids into these hydrogels enhances their mechanical properties and conductivity, positioning them as significant self-healing agents. This review focuses on the emerging field of stimuli-responsive ionic-based hydrogels and explores their potential in dermal applications and tissue engineering.

## 1 Introduction

Ionic gels are hybrid materials formed by dispersing ionic liquids within a polymer or inorganic matrix. This results in materials with high ionic conductivity in the solid state, making them suitable for various applications, including flexible electronics and biomedical devices.^1,2^ Ionic liquids (ILs), which are composed of organic cations and organic or inorganic anions, are salts that are present at nearly room temperature. They exhibit high thermal stability and negligible vapor at ambient temperature, contributing to their low flammability.^3^ The characteristics of ionic gels are influenced by the choice of cations and anions. Variations in anionic composition can lead to variations in the electronic environment of the cation, affecting the overall properties of the ILs. Commonly utilized cations in ILs include imidazolium, pyridinium, piperidinium, phosphonium and ammonium. Examples of anions include halides, carboxylates, sulfates, phosphates and various other species.^4^

Hydrogen bonding is crucial for affecting the reaction dynamics and viscosity and is essential for gelation, balancing gelator–gelator and solvent–gelator interactions. Electrostatic interactions between ILs and charge polymer networks enhance ionogel performance but can hinder ion transport and reduce conductivity. Solvophobic interactions cause nonpolar species to aggregate, improving the compatibility between ILs and polymers and influencing gelation. When polymers are added to the dispersion of ILs and nanoparticles, they form coils and absorb layers, leading to strong steric repulsion and nanoparticle clustering due to the bulky IL group.^3^ The incorporation of ionic liquids into a polymer matrix of ionogels enhances ionic conductivity by increasing the number of charge carriers and amorphicity while also acting as a plasticizer to improve ion mobility, although excessive ionic liquids can reduce mechanical strength and crystallinity. The purpose of ionic gels includes optimizing ionic conductivity for electrochemical devices, enhancing mechanical properties for flexibility and durability, and achieving self-healing capabilities. Additionally, tuning the responsiveness to external stimuli enables controlled drug release and adaptive behavior, while ensuring biocompatibility and biodegradability is crucial for biomedical applications.^5−7^ Ionic gels have attracted substantial interest because of their distinctive self-healing properties. Ionic gels can autonomously repair damage, recovering their original mechanical and functional characteristics after being subjected to stress or damage. Drug release from the ionogels was related to the gradual loss of ILs and internal loads. The suitability of ionogels formulated with biodegradable ILs for drug delivery applications has further increased.^4^

This self-healing capability is primarily due to the presence of multiple hydrogen bonds and dynamic interactions within the gel matrix, which enables the reformation of the network structure following damage.^8,9^ Recent advancements have produced various formulations of self-healing ionic gels that exemplify their unique capabilities. Zhang et al. developed a lignin/poly(ionic liquids) composite hydrogel dressing with exceptional self-healing properties, achieved through supramolecular interactions between lignins, which also improved the mechanical strength and antibacterial activity.^10^ Wang et al. fabricated Ag-lignin nanoparticles, polyurethane and ionic liquids.^11^ The self-healing capability was achieved through the introduction of disulfide bonds, with a self-healing efficiency of 97%.

In addition to the fundamental properties of ionogels, stimuli-responsive ionogels represent an advanced class of materials that respond dynamically to external stimuli. These innovative ionogels offer enhanced functionality, making them particularly suitable for applications requiring adaptability and self-healing properties. Stimulus-responsive polymers have undergone significant growth recently, attracting considerable attention from the academic and industrial sectors because of advancements in their synthesis and wide range of applications.^6^ Stimuli-responsive self-healing ionic gels are emerging as a transformative technology in the fields of dermal applications and tissue engineering. These innovative materials possess the unique ability to respond to external stimuli such as temperature, pH or light, enabling them to autonomously repair themselves after damage.^4^ Owing to their self-healing ability, biocompatibility, and tunable mechanical properties, these materials are ideal candidates for advanced therapeutic and regenerative applications. These gels can be categorized based on their matrix composition.

Ionic liquid-based gels are gaining attention in tissue engineering because of their versatile properties. They have been explored for applications such as artificial muscle and scaffolds.^12^ ILs such as [Bmim][Cl] and 1-ethyl-3-methylimidazolium trifluoromethanesulfonate [Emim][TFSI] enhance the mechanical properties and electrical conductivity of polyvinylidene fluoride (PVDF)-based electroactive films that support muscle cell proliferation.^13^ ILs also aid in dissolving proteins and polymers, such as silk fibroin keratin, collagen and cellulose, to form scaffolds for various tissue engineering applications, including bone and neural tissue repair.^12^ Hydrogels incorporating liquids have shown improved biocompatibility and functionality, such as providing cardiac tissue repair and skin generation, and have potential in cancer therapy.^12,14^ Overall, ionic liquid-based materials offer significant advancements in tissue engineering, with applications ranging from wound healing to cardiac patches. This review contains the fundamentals of ionic gels, their types and properties, various stimuli-responsive behaviors, the self-healing mechanisms involved and their applications in dermal and tissue engineering.

## 2 Fundamentals of Ionic Gels

### 2.1 Definitions and Characteristics

Ionic gels are advanced functional materials composed of dispersed phases containing ionic liquids (ILs) confined within a polymeric network.^4,15^ These materials integrate the properties of ILs and gels, enabling various applications. ILs are salts made up of organic cations and organic or inorganic anions. These exist as liquids below 100 °C. Gels, on the other hand, are three-dimensional (3D) networks containing colloidal particles dispersed in a liquid medium.^15,16^ In ionic gels, the ILs are confined within a 3D cross-linked network through different internal interactions, resulting in a customizable structure. The internal interactions involved in an ionic gel include hydrogen bonding, electrostatic interactions, steric interactions and solvophobic interactions. They are typically designed by forming polymer networks in an ionic medium, with the IL molecules being confined within the network of gelation.^4,17^ Ionic gels retain the properties of ILs, suggesting potential applications in wearable strain sensors and biochemical detection. The composition and type of an ionogel govern its properties. Ionic gels demonstrate high stability over time, even under reduced pressure, and retain the specific properties of the IL, except for outflow.^3,18^ The characteristic features of ionic gels include ionic conductivity, thermal stability, electrochemical stability and mechanical properties, among others.

### 2.2 Types of Ionic Gels

IL-based gels are classified into three types depending on their nature: ILs can serve as the continuous phase, the dispersed phase, or both phases simultaneously. These are known as poly(IL) hydrogels, ionic gels and IL gels. In poly(ILs) hydrogels, the ILs can be polymerized to form polymeric ILs (PILs) or copolymerized with other monomers to constitute the continuous phase. These gels are dispersed in an aqueous phase. In ionic gels, the continuous phase consists of non-PILs and ILs as the dispersed phase. The third type consists of IL gels, which involve the polymerization of ILs to produce PILs as a continuous phase, which are simultaneously dispersed in other ILs^15,19,20^

**Figure 1:**
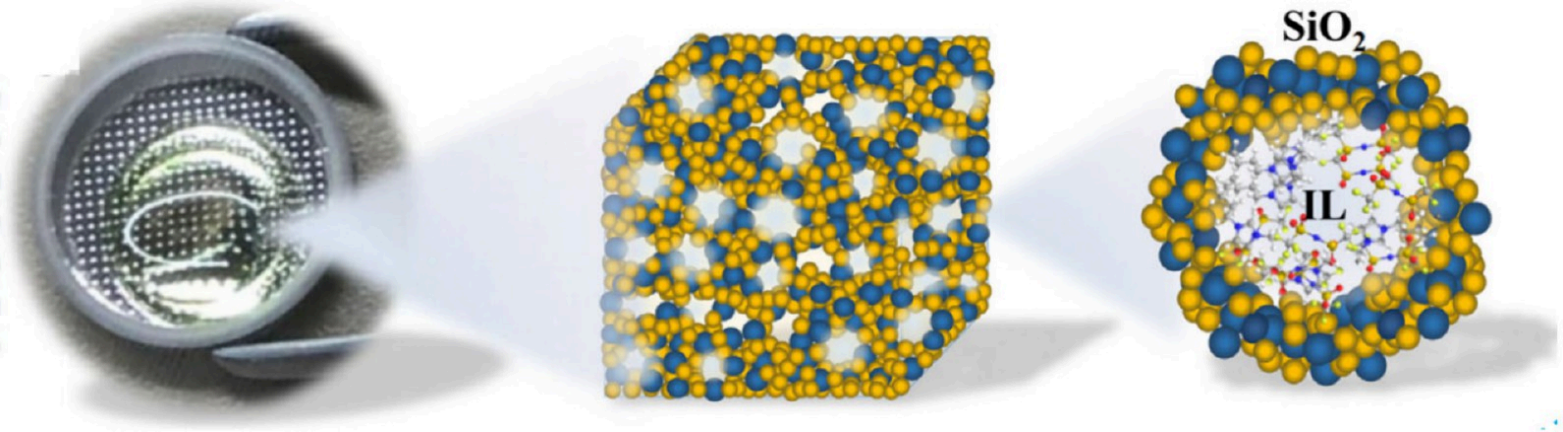
Photograph of the IE-T electrolyte and schematic of the internal microscopic three-dimensional structure of the ionogel. Adapted with permission from ref (26). Copyright 2019 Elsevier.

Ionic gels can also be classified based on the type of matrix: inorganic matrix, organic matrix and organic–inorganic hybrid matrix.^3^**Inorganic ionic gels** consist of ILs immobilized within an inorganic matrix with high ionic conductivity and structural stability.^21^ The inorganic ionic gels are further divided into bucky gels and silica-based ionogels. Bucky gels are formed by mixing ILs with carbon nanotubes, resulting in a dense suspension due to the gelification process.^22^ Silica-based ionogels consist of silica precursors and ILs.^23,24^ By forming a silica network or using silica as a nanofiller, the ionic conductivity is significantly improved. The silica network provides additional pathways for ion transport, while the nanofiller increases amorphicity, enhancing the mechanical properties.^3^ Khurana and Chandra synthesized silica-based ionic gels. The ionic gel was developed via the use of tetraethyl orthosilicate (TEOS) (a silica precursor), a poly(vinylidene fluoride-cohexafluoropropylene (PVDF-HFP) copolymer, lithium trifluoromethanesulfonate (LiTf) salt and EMIMTf as ILs.^25^ The nonaqueous method results in a high electrolyte solution content within the hybrid matrix, achieving a room-temperature ionic conductivity of 3 mS/cm, which is suitable for electrochemical applications. The ionic gel showed a wide electrochemical stability window, high thermal stability and increased amorphousness. A higher electrolyte content decreases the mechanical strength. Cheng et al. explored quasi-solid-state electrolytes with ionic liquids.^26^ Two ionogel quasi-solid-state electrolytes were synthesized by incorporating different lithium salts (LiOTf and LiTFSI) and a porous silica precursor (TEOS) with a [BMIM][BF4] ionic liquid. Both electrolytes have high ionic conductivity. The porous, cross-linked structures facilitate ion transport, overcoming leakage and flammability issues (Figure 1), making these ionic gel electrolytes promising candidates for use in lithium-ion batteries.

In another study by Wu et al., a novel solid-state ionogel electrolyte was fabricated using a silane coupling agent (SCA) to form a porous network that confines an ionic liquid electrolyte.^23^ Epoxy groups were grafted onto silica to enhance the ionic dissociation of Lt salts and the IL. The ionic gel with an optimal ILE/SCA molar ratio of 0.75 exhibited excellent thermal stability, a wide redox stability window and an ionic conductivity of 1.91 × 10^–3^ S/cm at 30 °C, surpassing the pure IL electrolyte.

The **organic ionic gels** consisted of a polymer matrix swollen with ILs. The backbone for organic liquids is typically formed from polymers such as polyacrylate, poly(vinyl alcohol), and elastomers, which provide structural integrity and mechanical properties.^27^ The organic ionic gels can be prepared by adding a low-molecular-weight gelator (LMWG) and gelation by an organic polymer. In LMWG, the LMWG (organogelator) is added to ILs at high temperatures to form a gel upon cooling. Additionally, this method uses cosolvents and results in high ionic conductivity. Cheng et al. tuned the ionic gel properties using LMWG, an amide-functionalized imidazolium-based surfactant, in various solvents and inorganic salt additives.^28^ Chemical (Cu+2/H_2_O_2_) and temperature stimuli induced the gel-to-sol phase transition. The addition of CuBr_2_ increased the mechanical strength and altered the gel morphology. In another study, Bielejewski and colleagues developed an ionic gel containing methyl-4,6-*O*-(*p*-nitrobenzylidene)-α-d-glucopyranoside (LMWG), which self-assembles in an aqueous solution of high-temperature ionic liquid tetramethylammonium bromide.^29^ Compared with pure liquid electrolytes, gel electrolytes exhibit enhanced ionic conductivity. These findings suggest that intermolecular interactions between ion complexes and gelator aggregates are responsible for the improved conductivity.

In the **gelation of ILs by organic polymers**, polymer ionic gels can be fabricated through three routes: (i) doping polymers with ILs, (ii) monomer polymerization in ILs, and (iii) polymeric ILs.^3^ Doping polymers with ILs involves blending or impregnating polymers with ILs to form ionic gels.^30^ Singh et al. studied the electrochemical, structural and photoelectrochemical properties of [EMIM][DCA] with PVDF-HFP.^30^ The inclusion of the [EMIM][DCA] IL enhances the charge carrier mobility, leading to increased ionic conductivity. The IL also introduces more amorphous regions within the polymer matrix, which is beneficial for conductivity, as confirmed by DSC and XRD measurements. Gupta et al. developed poly(ethylene oxide)-based polymer electrolytes containing lithium bis(trifluoromethylsulfonyl)imide and the ionic liquid 1-buty-3-methylpyridinium bis(trifluoromethylsulfonyl)imide.^31^ These electrolytes are thermally stable up to 360 °C. For a polymer electrolyte with 30% ionic liquid, the ionic conductivity was approximately 2.5 × 10^–5^ S/cm at 25 °C and 2.3 × 10^–4^ S/cm at 40 °C. The gel showed increased discharge capacity and decreased cell resistance at higher temperatures, which was attributed to improved electrode–electrolyte contact.

In the polymerization of monomers in ILs, the monomers are polymerized within ILs, producing flexible, self-standing films with high ionic conductivity.^32^ Marcinkowska et al. developed an ionic gel using a thiol–ene matrix formed from triallyl isocyanurate and trimethylopropane tris(3-mercaptopropionate) via photoinduced polymerization in ILs.^32^ The polymer matrix should have a phase-separated morphology and a colloid gel structure with interconnected microspheres. Polymerization was faster in ionic liquids with higher Kamlet–Taft beta parameters, whereas compatibility decreased with lower Kamlet–Taft alpha values. The ionic gel exhibited high ionic conductivity and retained most of the ionic liquid conductivity, demonstrating an antiplasticization effect due to the solubility of the ionic liquid in the matrix. In another study by Jiang and Company, free radical polymerization of methacrylate in the ionic liquid BMIPF 6 produced a new series of gel polymer electrolytes (GPEs) with high ionic conductivity.^33^ These GPEs were flexible, transparent and conductive with decreasing glass transition temperatures as the BMIPF 6 content increased the ionic conductivity of the GPEs, followed by the Vogel–Tamman–Fulcher equation, which reached nearly 10 at a power of 3 s/cm at room temperature, and the capacitor performance was assessed through cyclic voltmeter impedance spectroscopy with galvanostatic charging–discharging.

**Organic–inorganic hybrid matrix ionic gels** combine ILs, nanofillers and polymer matrices, enhancing the mechanical properties of inorganic fillers and the flexibility of organic polymers. These ionic gels are fabricated to address these issues and achieve ionic gels that are tough and have simple fabrication techniques.^34,35^ Su et al. developed an organic–inorganic semi-interpenetrating network.^36^ The ionic gel was fabricated by confining an IL with a cross-linked PIL copolymer and a glass fiber scaffold. This gel offers superior properties, including enhanced lithium and high mechanical strength, and the wire electrochemical window and fire resistance in tests allow over 1800 h of lithium plating or stripping without significant dendrite formation and demonstrated excellent cycling stability in full cycles with Li_3_V_2_ (PO_4_)_3_ ethyl. Lee et al. fabricated multifunctional ionic group-functionalized ladder-like polysilsesquixone to create hybrid ionogels with the ability to cross-link lithium-ion batteries in IL electrolyte media.^37^ The resulting ionogel electrolyte offers exceptional thermal stability, mechanical strength, high ionic conductivity and electrochemical stability.

### 2.3 Characteristics of Ionic Gels

**Figure 2:**
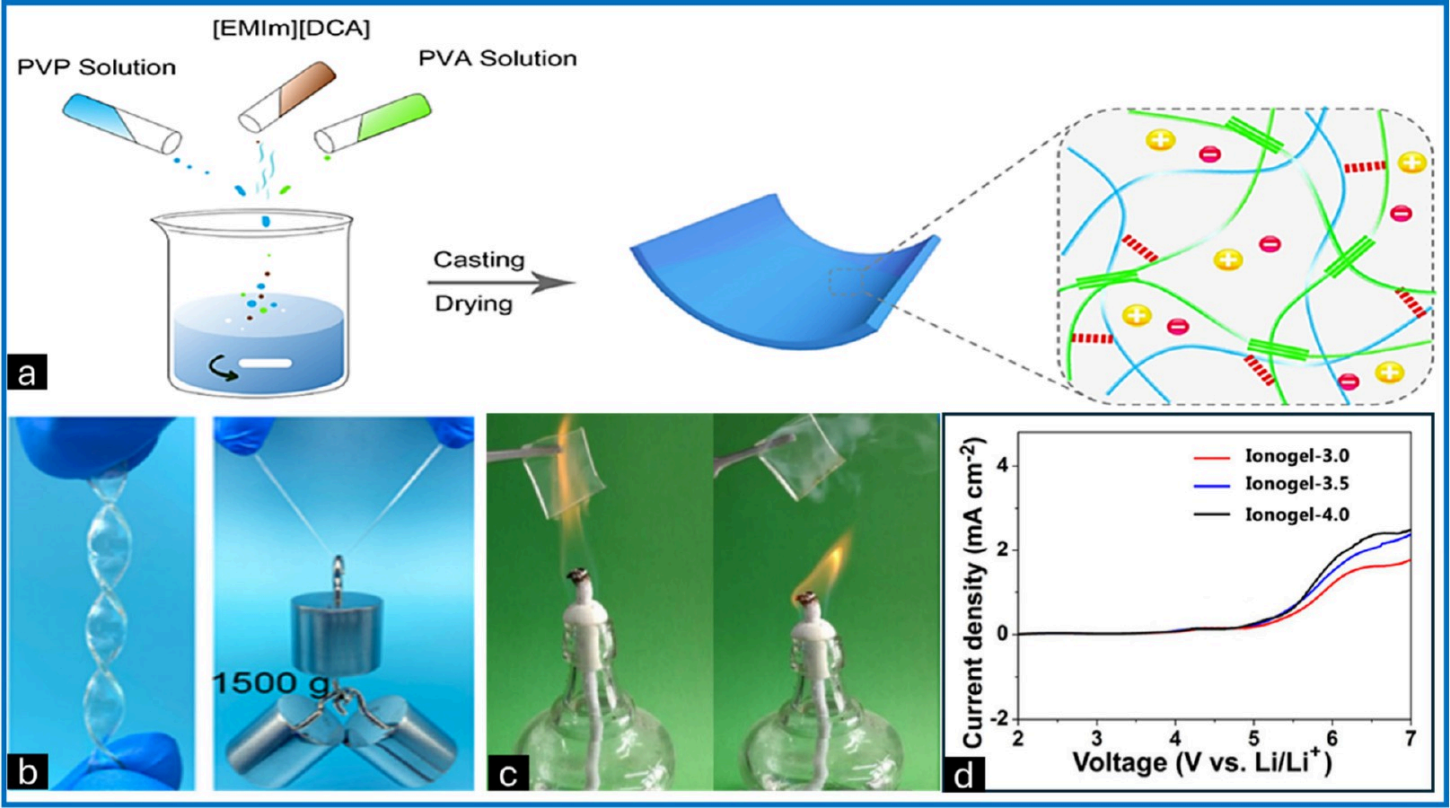
(a) Schematic representation of PVA/PVPm/ILn healable ionogels with good mechanical properties. (b) Images depicting the deformation of the PVA/PVP 0.4/IL2 ionogels (left) subjected to a load of 1500 g (right). (c) Images captured showing excellent flame retardation or thermal stability of the highly conductive organic-ionogel prepared from tributyl phosphate. (d) At 10 mV/s, linear sweep voltammograms of lithium|ionogel|stainless-steel cells with different electrolytes. Adapted from ref (49). Copyright 2020 American Chemical Society.

Ionic gels exhibit diverse properties depending on the specific IL and the gel matrix utilized. The general and fundamental properties of ionic gels are their pore size or porosity, mechanical strength, ionic conductivity, and electrochemical and thermal stability (Figure 2). Additionally, these gels may exhibit specialized properties such as biocompatibility, antibacterial activity, self-healing capabilities and luminescence.^4^

#### 2.3.1 Pore Size and Porosity

Pore size and porosity are essential determinants in the efficacy of ionogels for dermal or tissue engineering applications. The liquid condition of room temperature ILs is advantageous for the production of ionogels by infiltrating the pores of a 3D gel matrix with the respective IL. The pore dimensions of the solid networks within the gel are expected to affect the behavior of the bulky IL molecules confined within the pores.^38^ Typically, ILs can serve as a polymeric component in scaffolds inside ionogels by chemical bonding. Poly(ionic liquids), a category of ILs, can modulate the architecture of the ionogel scaffold and pore formation by facilitating controllable hydrogen bonding, electrostatic interactions, and host–guest interactions, or they may serve as a dispersing medium within ionogels, being encapsulated in the ionogel scaffold.^17,39,40^ Ionogels typically have pore sizes in the range of 7–12 nm.^41^ The exact pore size can vary depending on the specific composition and preparation method of the ionogel. For example, ionogels prepared using silica aerogels with choline dihydrogen phosphate IL have an average pore size of around 12.2 nm.^42^ Increased pore size and elevated porosity facilitate enhanced cellular infiltration and proliferation, which are crucial for tissue regeneration. This facilitates cellular movement through the scaffold, enabling successful tissue formation. Elevated porosity facilitates the movement of nutrients, oxygen, and waste products, which is essential for sustaining cell viability and fostering tissue growth. Notably, appropriate pore dimensions and porosity are crucial for angiogenesis within the scaffold, which is vital for the sustenance of newly developed tissues.^43^ Although increased porosity can enhance biological performance, it may compromise the mechanical integrity of the scaffold. Consequently, a balance must be achieved to guarantee that the scaffold is both biologically effective and mechanically stable. In line with this, conductivity plays a significant role in tissue engineering, by providing electrical stimulation and electrical signal transmission, thereby promoting and enhancing cell proliferation, differentiation, migration, and tissue regeneration.^44^ Larger pore sizes within an appropriate range result in increased conductivity. Ding et al. synthesized ionogels (PAMPS-[EMIM][DCA] (1-ethyl-3 methylimidazolium dicyanamide) gel) with varying pore sizes by adjusting the polymer content, discovering that ionogels with larger pore sizes exhibit superior conductivity.^45^

#### 2.3.2 Mechanical Properties

In practical applications, the mechanical properties of the gel are critical. Ionic gels prepared via covalent cross-linking typically exhibit enhanced mechanical properties, as they contain ILs and other polymers in the hydrogel network and interact through hydrogen bonding and electrostatic and hydrophobic interactions.^15,46^ The mechanical properties are typically evaluated via parameters such as fracture strain, fracture strength, Young’s modulus, and energy dissipation, which are all derived from the stress–strain curve. Additional critical properties include crack propagation resistance and antifatigue characteristics.^47^ Most ionic gels currently exhibit mechanical issues, including low strength, poor toughness, high crack sensitivity and susceptibility to fatigue damage. These strategies include the use of double networks, micro/nanocomposites, supramolecular interactions, multiscale structures, and phase separation.^18,47^ Rana et al. fabricated double network (DN) ionogel films with high ion conductivity, stretchability, and ultradurability.^48^ Compared with physically interpenetrating network (PIN) ionogels, the prepared DN ionic gels exhibited superior mechanical, thermal and electrochemical properties. Mechanically, they have higher tensile strength (3.2 MPa at 30 °C) and better energy dissipation (U_hys_ of 911 kJ/m^3^ at 30 °C and 250% strain) than PIN ionic gels. They also maintain significant strength and energy dissipation at elevated temperatures (1.4 MPa and 216 kJ/m^3^ at 100 °C). Compared with traditional gel electrolytes, DN ionic gels are stable for up to 300 s° and are nonflammable. DN ionic gels demonstrated enhanced ionic conductivity with increasing IL loading, stable performance under repeated stretching and high temperatures, and a broad electrochemical stability range (0–3 V), making them ideal for various applications. Wang et al. developed a multifunctional ionogel-based strain sensor using Ag-Lignin nanoparticles (Ag-Lignin NPs), polyurethane (PU) and ionic liquids.^11^ This gel exhibited excellent mechanical properties, such as tensile strength of 3.14 MPa and elongation at break ranging from 893 to 1241%. These properties are attributed to the plasticizing effect of the ILs and the dissolution of the crystalline regions, which increase the elongation at break. The ionogel also demonstrated a high self-healing efficiency of 97.6% due to reversible bonding and superior adhesiveness facilitated by Ag–Lignin NPs. Additionally, the ionogel possesses remarkable antifreezing, UV-shielding and antibacterial properties. They maintain their mechanical properties across a range of temperatures and exhibit high UV absorption and significant antibacterial activity against *E. coli* and *S. aureus*. As strain sensors, ionic gels display reliable performance, stable sensitivity and accurate real-time monitoring of human movement, highlighting their potential in various technologies.

#### 2.3.3 Temperature Resistance

Ionic gels have high thermal stability, and by modifying the structure of ILs, the thermal resistance of ionic gels can be adjusted to withstand both high and low temperatures.^47^ Mao et al. reported an ionogel electrolyte (PAAm/IL-X) composed of a polyacrylamide (PAAm) network and the IL 1-vinyl-3-methylimidazolium bis(trifluoromethylsulfonyl)imide ([VMIM] [TFSI]).^50^ The incorporation of [VMIM] [TFSI] enhanced both the thermal stability and conductivity of the electrolytes. The ionogel achieved up to 23 mS/cm conductivity at 90 °C, with 71% retention after 20 days at room temperature. The thermal decomposition temperature of PAAm/IL-X exceeds 220 °C, which is attributed to the presence of the IL and cross-linking structure, which enhances the thermal stability. The PEDOT/CC electrode, prepared by electroplating, demonstrates superior electrochemical performance, achieving a notable specific capacitance. The capacitance performance of ASSC-X increases by 5% at 90 °C, highlighting the potential of PAAm/IL-X as a safe electrolyte/separator for high-performance supercapacitors. In another study, Ren and colleagues developed IL-based click-ionic gels via thiol–ene click chemistry under mild conditions.^51^ These ionic gels retained excellent mechanical properties and resilience. They exhibited high ionic conductivity, transparency, and nonflammability across a wide temperature range (−75 to 340 °C) due to the presence of poly(1-butyl-3-vinyl imidazolium fluobutyl) (PIL-BF4), which has a low freezing point and high boiling point, preventing crystallization and maintaining elasticity at low temperatures. The combination of ionic and covalent cross-linking networks provides structural robustness, whereas strong hydrogen bonding between PIL-BF4 and polymer chains enhances mechanical strength and thermal stability. Compared with hydrogels that lost 75% of their weight in half an hour at 50 °C, IL-based gels remained stable with no weight loss even after 5 days at 250 °C, demonstrating their excellent high-temperature stability.

#### 2.3.4 Ionic Conductivity

**Figure 3:**
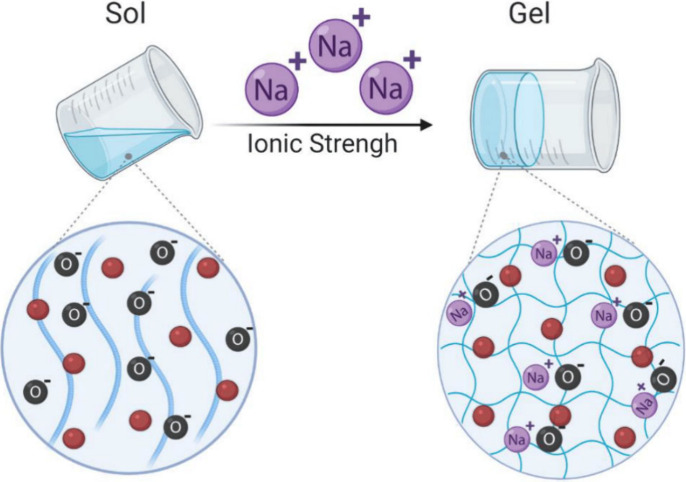
Schematic representation of the ionic-responsive hydrogel. An increase in the ionic strength caused increased hydrogel swelling. O, oxygen, Na^+^, sodium. The red circles represent hydrogel-loaded biomolecules. Adapted from ref (55). CC BY 4.0.

Ionic strength-sensitive gels change conformation in response to K^+^, Na^+^, and Ca^2+^ (Figure 3). They are made from ionizable and zwitterionic polymers such as alginate, deacetylated gellan gum, carboxymethyl dextran, poly(acrylic acid), and poly(itaconic acid).^52,53^ By increasing the swelling ratio proportionally to the ionic strength of the surrounding medium, poly(l-glutamic acid-*co*-l-lysine)-based hydrogels loaded with doxorubicin exhibited ionic strength sensitivity.^54^ Under high ionic strength, Cl^–^ and Na^+^ protect NH_3_^+^ and COO^–^ polypeptide groups, inhibiting electrostatic interactions and encouraging hydrogel expansion. The ionic conductivity of ionic gels is determined by ILs. However, the cross-coupled polymer networks restrict IL migration, resulting in a lower conductivity than that of pure ILs. The ionic conductivity of ionic gels is dependent on the viscosity and mobility of cations and anions in ILs and is influenced mainly by the chemical properties, size, viscosity of the ILs, the IL ratio, temperature and polymer matrix composition.^3,17,47^

**Figure 4:**
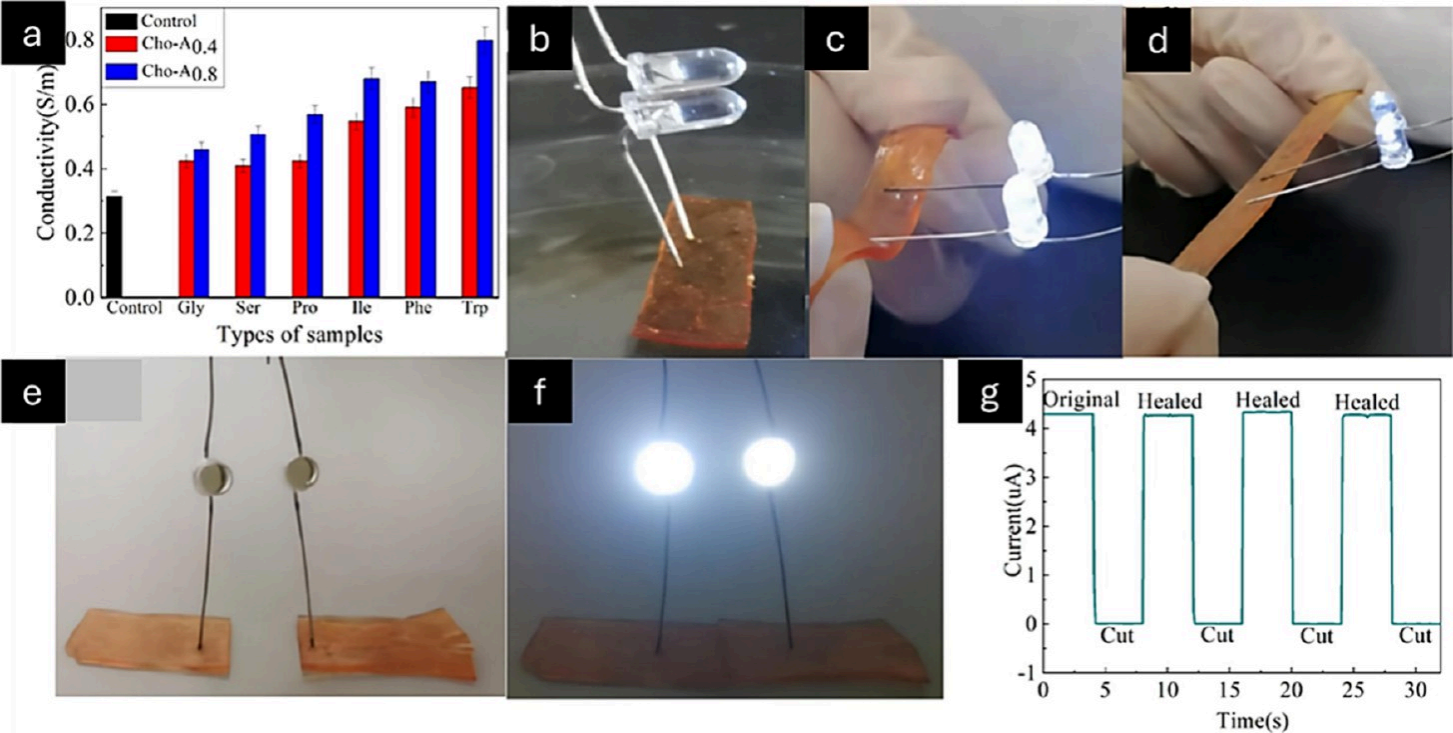
Conductive properties of hydrogels. (a) Conductivities of various hydrogels. (b, c) Images captured for the LED coupled with control and Cho-Trp0.8 hydrogel in sections. (d) Images captured for the LED linked to the elongated Cho-Trp0.8 hydrogel. (e, f) Images showing LED reaction to Cho-Trp0.8 hydrogel following incision and self-repair. (g) Recent modifications to the cutting and self-healing mechanisms of Cho-Trp0.8 hydrogel. Adapted with permission from ref (58). Copyright 2022 Elsevier.

Traditional electrolyte hydrogels have limitations such as low ion content, water evaporation, thermal stability and limited long-term storage; to address these challenges, alternative modifications have been explored.^16^ In the study conducted by Yan et al., a robust and highly conductive ionogel was fabricated by combining a DN structure, composite materials and high-functionality cross-linkers.^56^ The use of 1-ethyl 3-methylimidazolium dicyanamide ([EMIm][DCA]) and 1-butyl-3-methylimidazolium tetrafluoroborate ([BMIm]-[BF_4_]) as ILs and the charged PAMPS as the polymer network. The DN-PAMPS-GO ion gels exhibited high ionic conductivity, with the [EMIm][DCA]-containing gel achieving approximately 1.8 S/m and the [BMIm]-[BF_4_]-containing gel achieving approximately 0.28 S/m. These values are comparable to those of their respective neat liquids. The conductivity increases with temperature and remains stable over time, maintaining high performance even after 60 days. The addition of graphene oxide enhances the mechanical properties without significantly affecting the conductivity, making these ion gels ideal for flexible electronic and wearable devices. Weng et al. developed a PVA–PVP complex and [EMIm][DCA]-containing ionogel (PVA/PVPm/IL ionogel).^49^ The ionogel demonstrated high performance in terms of ionic conductivity and mechanical properties. The ionic conductivity of these ionic gels increases with temperature and IL content, reaching 19.7 mS/cm at room temperature, due to the high intrinsic conductivity of [EMIm][DCA] and the compatibility between [EMIm][DCA] and the PVA–PVP complex. Additionally, the ionic conductivity increases with increasing temperature, as higher temperatures increase the flexibility of the polymer chain and the mobility of the IL, leading to better ion movement within the matrix. Additionally, the ionogel exhibited good mechanical properties, with tensile stress and strain values varying for the IL and PVP contents. The mechanical strength decreased with increasing IL content due to the reduced density of the polymer network. By varying the molecular weight of the polymer, the electrochemical stability window can be varied. These properties make ionic gels suitable for high-performance, flexible electronic applications. A new series of hydrogen with ionic conductivity based on choline amino acid polyionic liquids was developed via double network methodology by He et al.^57^ These conductive ionic gels demonstrated excellent mechanical properties, self-healing ability and antimicrobial activity. The conductivity of Cho-Aa hydrogels was markedly superior to that of the control hydrogel, ranging from 0.409 to 0.798 S m^–1^ (Figure 4). Furthermore, the conductivity was augmented with the rising concentration of PILs. The incorporation of PILs exhibiting high ionic conductivity contributed to the enhancement of hydrogel conductivity. The network of Cho-Proa, Cho-Ilea, and Cho-Phea hydrogels was less dense than that of Cho-Glya and Cho-Sera hydrogels, facilitating the flow of ions within the hydrogel network. Consequently, Cho-Proa, Cho-Ilea, and Cho-Phea hydrogels showed enhanced conductivity. The superior conductivity of Cho-Trpa hydrogels, compared to other hydrogels, is attributed to the synergistic effects of ion migration and π–π electronic transitions of indole groups in tryptophan molecules.

#### 2.3.5 Electrochemical Stability

The electrochemical stability of ionic gels is crucial for their performance and is influenced by their composition, structural integrity, and methods used for assessment. ILs provide a wide electrochemical stability window, enhancing the stability of ionic gels for electrochemical applications.^4,16^ This stability, reaching a potential of up to 5.4 V, is determined by the oxidation and reduction potential, which must exceed the electrochemical potential of both the cathode and anode and fit within the conduction band maximum (CBM) and valence band minimum (VBM) of the electrolyte.^3^ The structure and composition of ILs, particularly the alkyl chain length and functional groups, also influence the stability and suitability of ionic gels across a wide temperature range.^3^ Li et al. developed ionic skins (I-skins) by impregnating poly(urea-urethane) (PU) with the ILs 1,2-dimethyl-3-ethoxyethylimidazole bis(trifluoromethanesulfonyl)imide ([DEIM][TFSI]).^59^ This combination showed excellent results with properties similar to those of human skin. The PU-IL2 ionic gel-based I-skins demonstrated high ionic conductivity (1.2 mS/cm), excellent electrochemical stability, high sensitivity to a wide range of strains (0.1--300%) and pressures (0.20 kPa) and maintained performance over 10,000 strain cycles and 200 days of open-air storage. In another study by Han et al., an ionogel containing a polyurethane acrylate (PUA) oligomer and 1-ethyl-3-methyl imidazolium bis(trifluoromethylsulfonyl)imide (EMITFSI) was fabricated under UV conditions.^60^ The results revealed that the electrochemical stability window increased with increasing PUA content, increasing the stability but reducing the ionic conductivity. Conversely, a higher EMITFSI content improved the ionic conductivity but decreased the electrochemical stability.

## 3 Stimuli-Responsive Behavior

ILs within the gel matrix make up ionic gels, which exhibit properties depending on their composition, the ratio used, the matrix material and the interactions between the components.^61,62^ The stimulus-responsive behavior of ionic gels represents a cutting-edge area of research that merges the properties of ionic liquids with those of polymeric networks, enabling these materials to undergo significant changes in response to various external stimuli. These behaviors are characterized by their ability to change physical or chemical properties in response to external stimuli, such as temperature, pressure, magnetic signals, light, and pH.^5^ This responsiveness makes ionic gels particularly valuable for a variety of applications.

### 3.1 Physical Responsive Stimuli

Ionic gels are capable of undergoing significant changes in their physical state in response to various external stimuli, such as temperature, pressure, light, magnetic field, and strain, which aid in improving their performance. Ionic gels are versatile in applications such as drug delivery, sensors and actuators because of their sol–gel transition, enabling precise therapeutic release and responsive material design for adaptive environments^1,5^

#### 3.1.1 Temperature-Responsive Stimuli

**Figure 5:**
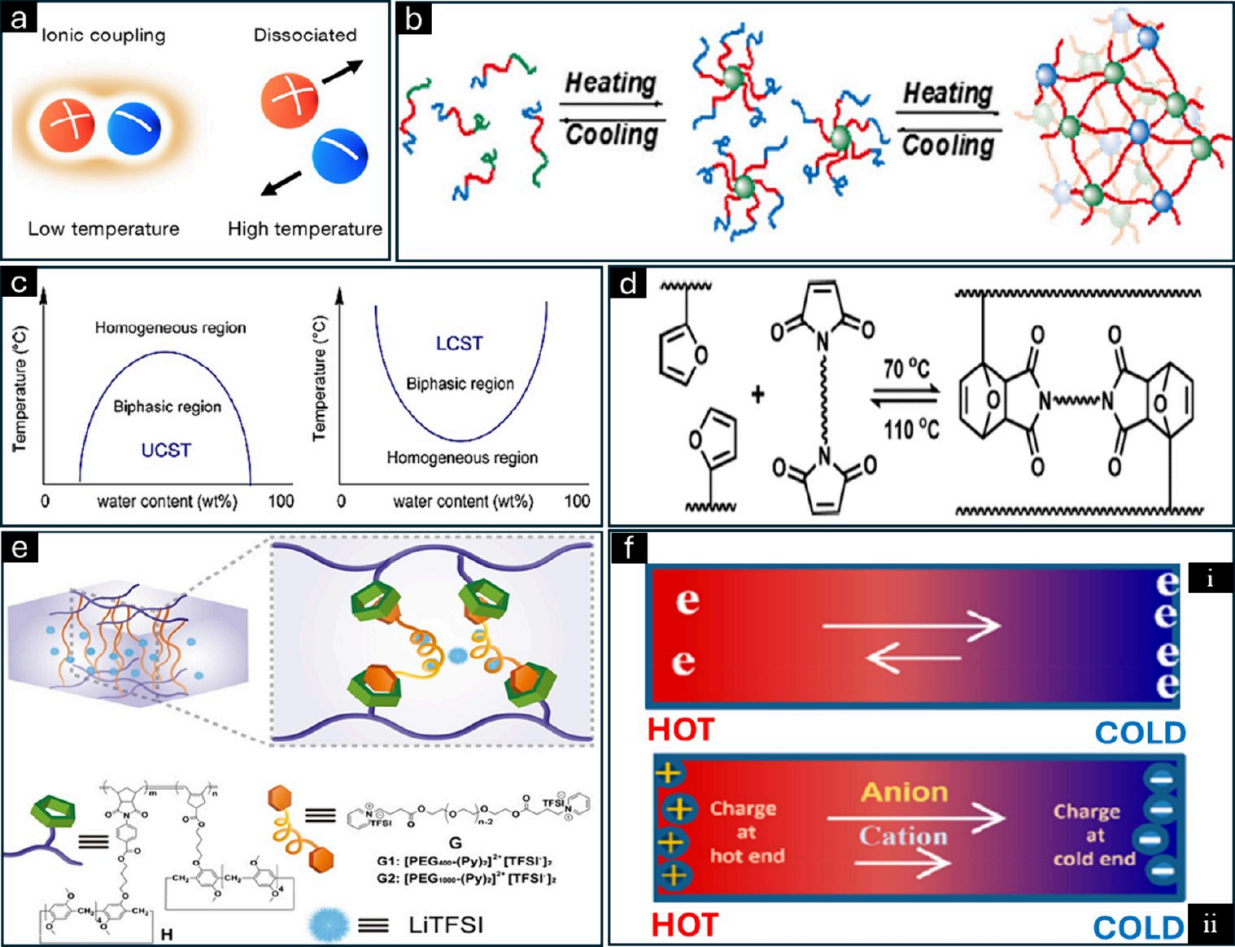
(a) Temperature-responsive mechanisms of ionic gels: influence of temperature on ionic pair decoupling-coupling. Adapted from ref (76). Copyright 2020 American Chemical Society. (b) Sol–gel process of the ionic gels. Adapted from ref (77). Copyright 2016 American Chemical Society. (c) Illustration of the upper critical temperature and lower critical temperature. Adapted from ref (78). Copyright 2015 American Chemical Society. (d) Influence of temperature on dynamic covalent bonding. Adapted from ref (79). Copyright 2018 American Chemical Society. (e) Supramolecular ionic gels constructed by host–guest complexation. Adapted from ref (80). Copyright 2024 American Chemical Society. (f) Seebeck and Soret effect cations and anions in an n-type electronic material subjected to a temperature gradient. The arrows in (i) illustrate that electrons migrate in two distinct paths under a temperature gradient, with more electrons migrating toward the cold end than toward the hot end. The two arrows in (ii) indicate the migration of both cations and anions from the hot end to the cold end, with anions migrating at a faster rate than cations. Adapted from ref (81). Copyright 2022 American Chemical Society.

Temperature-responsive ionic gels control the transport of ions by changing their charge density in response to temperature.^63^ The mechanisms involved in the temperature behavior of ionic gels include ion pair decoupling-coupling, sol−gel transitions, critical temperature effects, temperature-responsive dynamic covalent bonding, supramolecular response mechanisms and the Soret effect (Figure 5).^5^ The influence of temperature on an ion pair dissociation and coupling is that at room temperature, most of the cations and anions in the ILs of ion gels are neutral ion pairs, with few dissociative ions free to migrate. However, with increasing temperature, electrostatic interactions between anions and cations occur, and freely flowing cations and anions are formed (Figure 5a).^64^ The dissociation affects the sensor’s impedance, phase angle and capacitance in the temperature range of 30–80 °C. The results from the study revealed that as the temperature increased, the impedance decreased significantly, possibly because of enhanced ionic dissociation and increased charge carriers, which in turn improved the ionic conductivity. The increased capacitance with temperature was attributed to the accumulation of decoupled ions at the interface between the ILs and the electrode, forming an electric double layer. The phase angle was decreased in the low-frequency regime because of the increased electrical double-layer capacitance. At high frequencies, the ionic conductivity led to a decreased phase angle. Sastry and Singh synthesized an amphiphilic room-temperature ionic liquid, 1-dodecyl-1-methylpiperidinium acetylsalicylate [C_12_mpip]-[AcSa], from [C_12_mpip] via ion exchange.^65^ The critical aggregation concentration (CAC) of [C_12_mpip]-[AcSa] was lower than that of the chloride precursor, indicating stronger interactions between the cation and the bulky acetylsalicylate anion as well as enhanced hydrogen bonding with water. Rheological studies have shown that the addition of sodium salicylate transforms small ellipsoid aggregates into large worm-like structures with viscoelastic gel properties. The gel network exhibited complex temperature-dependent moduli without hysteresis, and the release of the acetylsalicylate anion was triggered by dilution, leading to surface erosion and demicellization.

The sol–gel transition mechanism takes place in certain polymer/IL blends. Here, the polymer dissolves in the IL, resulting in micelles, which self-assemble to form a gel upon heating (Figure 5b).^62^ A study conducted by Kitazawa and co-workers investigated morphological modifications and concomitant sol–gel transitions using a doubly thermosensitive ABC-triblock copolymer in an IL.^66^ The gelation behaviors of poly(benzyl methacry-late) (PBnMA), poly(2-phenylethyl methacrylate) (PPhEt-MA) and poly(methyl methacrylate) (PMMA) were studied, revealing a sol–gel transition above certain concentrations. At 20 wt %, the dynamic moduli shifted from a solution at low temperatures to a gel at high temperatures. However, gelation occurred at a lower temperature than expected, suggesting that the system forms a jammed micelle state, not a polymer network, due to micelle jamming at intermediate temperatures. For the 10 wt % polymer solution, gelation occurred at 130 °C, indicating that the critical gelation concentration lies between 10% and 20%. The high critical concentration is attributed to the low glass transition temperature of PBnMA, which affects the toughness of the cross-linking points and the retention capacity of the IL in the polymer network.

Ionic gels exhibit a critical temperature effect, in which the materials undergo a phase transition at a specific temperature, which is further divided into the lowest critical solution temperature (LCST) and upper critical solution temperature (UCST) depending on the temperature (Figure 5c). The thermoregulatory properties of ILs are influenced primarily by electrostatic and hydrogen bonding interactions.^67^ Ionic gels containing polymers such as poly(*N*-isopropylacrylamide)^68^ exhibit an LCST phenomenon in water, with a phase transition temperature. Above the LCST, the ion gel and water undergo phase separation. This increase in temperature caused an increase in the ion concentration within the gel, leading to increased ionic conductivity. Similarly, the polymers in ionic gels exhibit UCST behavior, where they undergo a phase transition from a swollen state to a shrunken state with decreasing temperature. Polymers such as poly(vinyl methyl ether) (PVME), poly(vinyl alcohol) (PVA) and poly(2-hydroxyethyl methacrylate) (PHEMA) display both UCSTs and LCSTs, although the UCSTs are below the freezing point of normal pressure water.^69^

In temperature-responsive dynamic covalent bonding, gels are composed of polymers linked by dynamic covalent bonds that can break and reform in response to changes in temperature (Figure 5d).^70^ Tang et al. fabricated thermally healable DN ionic gels containing poly(furfuryl methacrylate-*co*-methyl methacrylate) (P(FMA-*co*-MMA)) and poly(vinylidene fluoride-*co*-hexafluor-propylene) (P(VDF-*co*-HFP)).^71^ This design was chosen because gels with thermally reversible dynamic covalent furan–maleimide bonds transition from a static state to a dynamic state with increasing temperature, enabling thermal healing. At 70 °C, the dynamic covalent bonds in the ionic gels are cross-linked, enhancing their mechanical properties. As the temperature increased to 110 °C, these bonds broke, weakening the mechanical strength of the ionic gel. In the study by Hall et al., the process of thermoreversible gelation of a triblock terpolymer known as poly(ethylene-*alt*-propylene)-*block*-poly(ethylene oxide)-*block*-poly(*N*-isopropylacrylamide) (PON) in the presence of the ionic liquid, 1-ethyl-3-methylimidazolium bis(trifluoromethyl sulfonyl)imide ([EMI][TFSI]) was reported.^72^ The use of small-angle X-ray scattering, rheology, and dynamic light scattering revealed that PON ionic gels have a wider sol–gel transition. The expected rheological behaviors were achieved at the point of gelation and as viscoelastic fluids at high temperatures. With 10 wt % polymer loading, the network had 98% elastically effective strands. This allowed the gel to withstand stresses of up to 70% at 0 °C. This was attributed to the distinct thermoresponsive phase behavior of poly(*N*-isopropylacrylamide) in ionic liquids and water. Overall, the results concluded that the PON system generates a more efficient gel network, allowing lower polymer concentrations.

The supramolecular response mechanism involves noncovalent interactions. Qin et al. fabricated pillararene-containing polymers with various backbones to study the effect of the polymer structure on the conductivity (Figure 5e).^73^ Different polymers were created by the polymerization of pillar[5]arene-functionalized norbornene monomers (NMP5A) and 1,6-pyridyl monomers (DYP5A). The supramolecular interactions occur primarily between the pendant pillararenes in the polymer (host H1–H5) and the pyridinium moieties in the guest molecules. These polymers formed complexes with dipyridinium-functionalized poly(ethylene glycol) ILs (G1 and G2) through host–guest interactions, forming supramolecular IL gels. The conductivity of the gels was studied at 30, 40, 50, and 60 °C. The H3-G1 system conductivity decreased upon the addition of LiTFSI, whereas the H3-G2 system conductivity increased. This difference is attributed to the physical state and number of EO units in PEG400 and PEG1000.

The Soret effect involves the thermal diffusion of ionic components; when an ionic gel is subjected to a temperature gradient, cations and anions accumulate at the hot and cold ends due to the Soret effect, which creates a voltage difference that can be used to determine the ambient temperature range (Figure 5f).^5,74^ The migration rates of anions and cations in ionic components differ due to variations in their volume and functional groups, leading to concentration differences at the cold and hot ends. This results in a voltage difference between the ends, which can be used to determine the ambient temperature range.^75^

#### 3.1.2 Pressure-Responsive Stimuli

To determine the pressure-responsive mechanism, the device design is more important for realizing a sensitive pressure response than the influence of the structure and properties of ionic gels. The different types of pressure response mechanisms involve piezoresistive, piezoelectric, capacitive and triboelectric mechanisms.^5^ In the piezoresistive mechanism, when pressure is applied to an ionic gel, the ions within the gel structure are displaced, causing changes in the electrical properties that can be detected as a pressure signal. The sensitivity and detection range of the piezoresistive response can be tuned by adjusting the composition and stiffness of the ionic gels, which provides high sensitivity at low pressure. Zhang et al. fabricated ionic gels via *in situ* synthesis of 1-vinyl-3-ethylimidazolium dicynamide ([VEIm][DCA]) ILs containing a carbon–carbon double bond.^82^ The electrical properties were measured via a Keithley 4200 semiconductor meter. The sensor responded to changes in pressure and exhibited high sensitivity, a low detection limit and a rapid response with slight pressure changes.

In the piezoelectric response, materials can generate an electric charge in response to the applied mechanical stress and vice versa.^83^ They respond to low frequencies and offer isotropic electromechanical responses. These materials exhibit polarization when subjected to intense electric fields, a process known as poling. In a study conducted by Villa et al., barium titanate (BaTiO_3_) nanoparticles were fabricated into a gel matrix with an IL, creating a material that responds to low-frequency mechanical stimuli with improved output voltages and anisotropic behavior.^84^ The gel retained elastomeric-like properties, which are beneficial for low-frequency electrochemical response along with mechanical stability.

The pressure response concerning the capacitive mechanism refers to the ability of a gel to change its capacitance in response to applied pressure. Zhang and colleagues developed a capacitive flexible pressure sensor for positive and negative pressures.^85^ The flexible sensor demonstrated high sensitivity, with negative and positive pressures of 84.45 nf/kPa and 25.61 nf/kPa, respectively. It maintained stability over 100 pressure cycles and remained functional under bending, with response times of approximately 250 ms.

#### 3.1.3 Light-Responsive Stimuli

**Figure 6:**
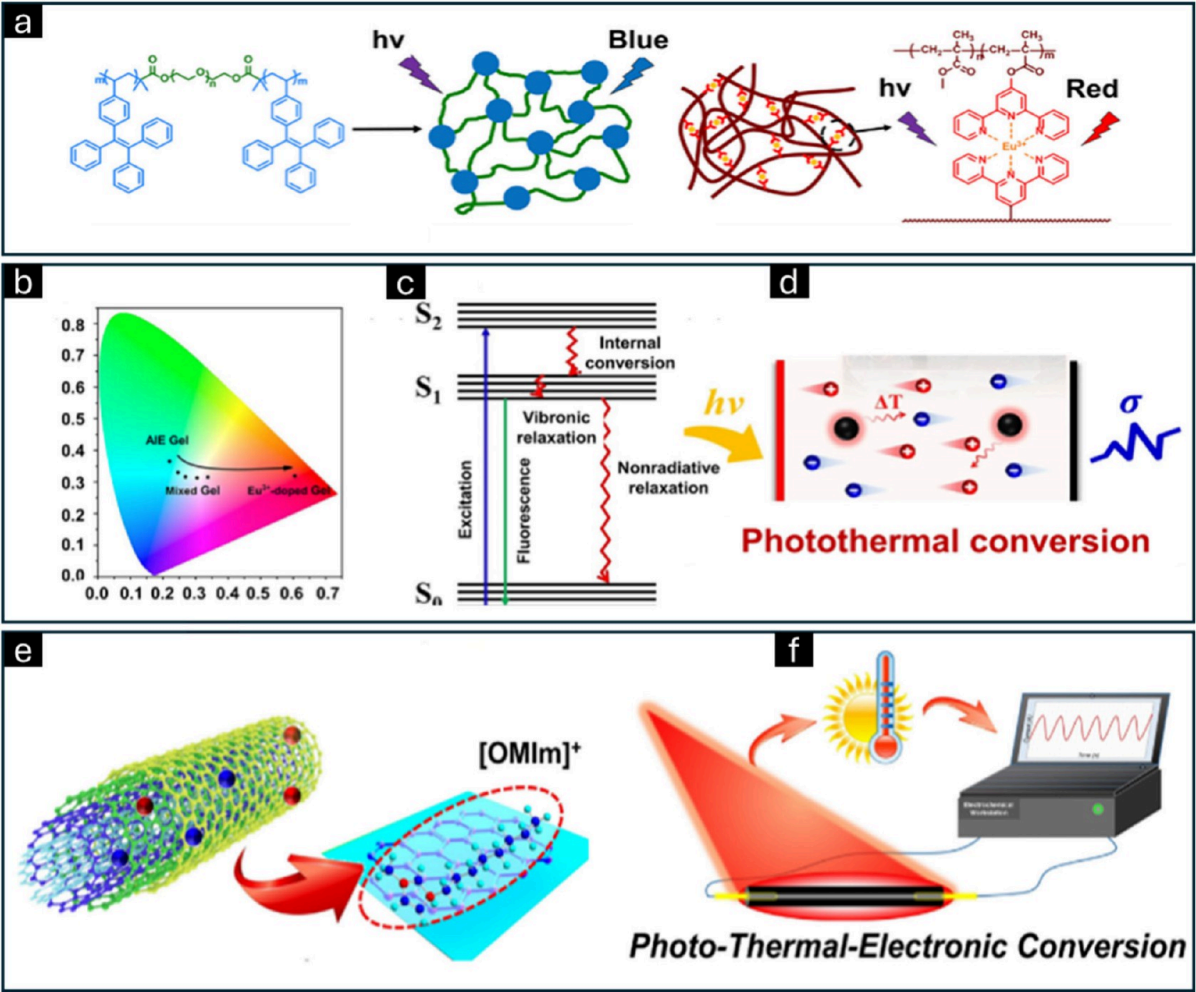
(a) Polymer backbone functionalized with light-emitting moieties. (b) Tuning of the luminescence spectrum through variation in the mixing ratio of these luminescent groups. (c) Mechanistic insights into the photothermal conversion process. (d) Mechanism underlying the light-responsive behavior of PIL. (e) Interaction dynamics between carbon nanotubes and ILs. (f) Schematic representation of the photothermal conversion mechanism of ion gels. Adapted from ref (88). CC BY 4.0.

Ionic gels respond to light in addition to temperature and pressure signals and have applications in the biomedical industry. The incorporation of light-responsive ILs can impart these properties to ionic gels. The different light-responsive mechanisms involved include aggregation-induced emission (AIE), photothermal substances and inorganic nanoion composites (Figure 6). Yang et al. synthesized AIE-based poly(IL) containing 4-(1,2,2-triphenylvinyl)phenyl acrylate (TPE), which is a polymeric material whose fluorescence is enhanced upon aggregation or solid-state formation. TPE showed a synergistic effect on AIE-based poly(ionic) liquids with enhanced quantum yield.^86^ Zhang et al. developed a hydrogel actuator that is responsive to multiple stimuli.^87^ The actuator was made of poly(*N*-isopropylacrylamide) coated with a spiropyran moiety, which is a photochromic and hydrophobic molecule. This combination allowed the gel to exhibit reversible bending behaviors driven by changes in temperature and solvent, as well as the ability to form patterns in response to light. The isomerization of spiropyran during Vis/UV light irradiation altered both hydrophobicity/hydrophilicity and resulted in distinct fluorescence characteristics. Due to the photoisomerization of spiropyran, the PNIPAAm-SP hydrogel exhibited photoswitchable “on/off” fluorescence activity. When irradiated with white light in H_2_O, the gel displayed mild fluorescence emission at 610 nm, primarily due to the fluorescence produced by the ring-closing form; under UV illumination, the gel exhibited a pronounced emission at 665 nm, characterized by intense red fluorescence. Following eight cycles of UV/white light irradiation, the fluorescence intensity at 665 nm of the gel exhibited no significant alteration, demonstrating the gel’s remarkable reversibility and resistance to fading.

ILs containing UV or light-responsive materials exhibit changes in their properties upon exposure to UV or visible light. These gels can be prepared by physically mixing ILs with light-sensitive compounds. Ionic gels with a UV response can emit blue fluorescence at a wavelength of 480 nm.^5,16^ A study conducted by Patel et al. formulated photoresponsive ILs into a polymeric hydrogel. This study aimed to develop a photoresponsive ionic gel with good mechanical properties, thermal stability, a thermoresponsive reversible transition into a sol form, and the ability to convert it into a sol form only through light irradiation.^89^ The hydrogel containing [C8EMorph][MO], which has photoresponsive characteristics, exhibited significant photoresponsive behavior when irradiated with light at a wavelength of 460 nm. When the IL concentration exceeds its critical micelle concentration (CMC) of 8.0 mM, the hydrogel transitions from a gel to a sol after prolonged light exposure. This transformation is irreversible and occurs without a significant increase in temperature. Below the CMC, no photoresponsive behavior was observed. SEM images revealed that the hydrogel’s 3D fibrous network was disrupted upon irradiation, indicating that the IL azobenzene group underwent trans-to-cis isomerization, altering its conformation and breaking the hydrogel structure. The rheological measurements indicated that the hydrogel is stable and exhibits viscoelastic behavior up to certain critical strain levels and angular frequencies, and the hydrogel displayed a temperature-dependent gel–sol transition. The hydrogel exhibited distinct and irreversible photoresponsive behavior due to the trans-to-cis isomerization of [C8EMorph][MO] under light irradiation, resulting in a gel-to-sol transition. Additionally, it demonstrates reversible thermoresponsive behavior, transitioning between gel and sol states with temperature changes.

The other method involves incorporating nanoion composites of ionic gels, which enable an effective photothermal response, leveraging their high photothermal conversion efficiency. In the study conducted by Gao et al., a system containing photosensitive ILs and carbon nanotubes was fabricated.^90^ The photosensitive IL was developed by dispersing carbon nanotubes in 1-octyl-3-methylimidazolium chloride ([OMIm]Cl), increasing their light absorption and photothermal properties. The system demonstrated remarkable stability, conductivity and thermal stability, making it highly effective for light detection and solar tracking applications.

#### 3.1.4 Magnetoresponsive Stimuli

Magnetic ion gel materials are fabricated by incorporating magnetic substances into a polymer matrix or by using magnetic ionic liquids.^1^ Shojaee et al. fabricated magnet-responsive ionic gels containing carbomers and choline hydroxide.^91^ The catalytic performance of the magnetic ionogel was assessed via a one-pot condensation reaction between benzaldehydes, dimedone and malononitrile in water. It achieved a 68% yield at room temperature in 4 h, which improved to 96% in 1 h at 60 °C. Solvent effects were studied, with only water proving effective. Increasing the ionogel amount to 100 mg did not affect the yield or time, whereas reducing it to 10 mg required a longer reaction time. The temperature impacts the reaction kinetics and equilibrium, shifting toward higher yields at elevated temperatures. Kulshrestha et al. developed a magnetoresponsive hydrogel by combining gelatin with a valine-based magnetic ionic liquid surfactant [ValC16][FeCl_4_] without nanoparticles.^92^ The biocompatibility was confirmed through interactions with animal DNA, as evaluated by circular dichroism (CD), zeta potential, ethidium bromide assay and agarose gel electrophoresis. CD spectroscopy indicated that [ValC16][FeCl_4_] maintains the DNA structure up to 0.7 mM but causes compaction at higher concentrations. Zeta potential measurements and EB exclusion assays confirmed the formation of the DNA-surfactant complex. Agarose gel electrophoresis revealed no DNA degradation at lower concentrations, with charge neutralization at higher concentrations.

#### 3.1.5 Ultrasound-Responsive Stimuli

Ultrasound (US) is a potent external stimulation, utilized as needed, to facilitate the controlled release of drugs from gels to a targeted site within the body. The efficacy of these systems is ascribed to cavitation, which occurs due to the alternating expansion and contraction of gas-filled microbubbles caused by the pressure fluctuations produced by ultrasonic energy. Ultimately, these cavitating microbubbles collapse, producing localized shock waves that may disturb nearby polymer assemblies.^93^ Ionic gels that react to the US have a variety of possible uses in the biomedical industry. When subjected to ultrasonic vibrations, these gels, which are usually made of ILs, may change physically. Ye et al. reported the fabrication of multifunctional visualized electronic skin (e-skin) based on the novel poly IL-ionogel.^94^ The translucent and conductive poly-IL ionogel (P(AAm-IL)) was synthesized through the photopolymerization of acrylamide (AAm) with 1-vinyl-3-butylimidazolium tetrafluoroborate (IL) in ethylene glycol (EG). The preparation of ionogel was attained using magnetic stirring and US treatment. The fabricated ionogel demonstrated superior stretchability (>4300%), enhanced conductivity (1.44 × 10 S·m^–1^), commendable transparency (>80%), and an extensive operational temperature range (20 to 80 °C) The luminous e-skin displayed both highly sensitive electrical responses and observable fluorescence alterations in reaction to external strain fluctuations. Rizzo and co-workers investigated the gelation properties of cyclodextrins and dicationic imidazolium salts. They also examined the self-healing capabilities of these gels when exposed to external stimuli, such as magnetic stirring or US irradiation.^95^ The qualities of the resultant soft materials in gel formation were examined by assessing their thermodynamic stability, analyzing their optical characteristics by UV/Visible spectroscopy, and investigating their shape via scanning electron microscopy. The obtained data indicated that the characteristics of these two-component gels could be regulated by altering the kind of cyclodextrin, the salts, or, more straightforwardly, by adjusting the host/guest ratio. Yan et al. performed experiments to assess the self-repairing properties of gels by magnetic stirring or US irradiation.^96^ All gel phases exhibited a good response to mechanical stimulation. Billeci et al. synthesized organic salts using diimidazolium and dipyrrolidinium ions to formulate gels in both organic solvents and ILs.^97^ These materials displayed sonotropic behavior and revealed self-repairing capabilities upon exposure to US irradiation. Upon comparison of the data from pyrrolidinium and imidazolium salts, it was apparent that the aromatic salts had a more favorable reaction to both magnetic stirring and US irradiation. The effect of US on the structural integrity of the fabricated hydrogels was reported by Li and his colleagues and the results showed that US obliterated the gelatinous matrix between PVA, and IL ([BMI]Cl).^98^ FT-IR data indicated that the intensities in the OH stretching were amplified following US exposure. The results indicated that US exposure could disrupt the hydrogen bonds in the PVA/[BMI]Cl composites, with the hydrogen bonds produced by adjacent OH groups, OH groups in disparate PVA chains, and the OH group interacting with the Cl– anion being readily affected by ultrasound exposure.

Guo et al. demonstrated a US-induced reversible sol–gel transition approach driven by modified noncovalent interactions.^99^ The resulting gels exhibited self-healing properties and tunable emission colors upon the incorporation of inorganic ions into the gel matrices. Through a heating–cooling process, the gel reverted to a sol state. Concurrently, a vesicle-tube morphology transition, governed by sonication and heating–cooling, was observed, alongside the aggregation-induced emission enhancement (AIE) property of the gel. The findings indicated that the US facilitated the J-aggregation of terpyridine motifs and augmented the hydrogen bonding interactions of TEC molecules, thereby initiating the gelation process. Hydrogel scaffolds serve as delivery vehicles for regenerative growth factors in tissue engineering. US has been investigated as a stimulus for attaining spatial and temporal control with responsive scaffolds due to its potential for translatability of the interactions of US with droplets and related bubbles in sonosensitive hydrogels.^100^ The acoustic droplet vaporization (ADV) and inertial cavitation (IC) thresholds were influenced by alterations in acoustic responsive scaffold (ARS) parameters, including fibrin concentration, emulsion shell material, PFC core, emulsion structure, and the number of acoustic cycles. ADV transpired within an ARS with negligible impact on cell viability, whereas IC resulted in diminished viability. This study recommended an ARS composition for growth factors (GFs) administration including 5 or 10 mg/mL fibrin with a double emulsion containing PFH or a mixture of PFP/PFH. For US exposure, an increased number of acoustic cycles was also advised. Notably, studies by Huang et al. showed the influence of ILs (1 mg/mL 1-butyl-2,3-dimethyl imidazolium chloride) and US on the enhanced solubility of soy protein isolate to 71.8%, thereby modifying the microstructure of the globular proteins.^101^

### 3.2 Chemical Stimuli

The majority of ILs include quaternary ammonium structures and are sensitive to gases such as carbon dioxide, hydrogen sulfide and ammonia.^5^ Ionic gels containing imidazolium show reversible sol–gel transitions in interactions with CO_2_. In the study conducted by Zhang and colleagues, a CO_2_-responsive ionic gel was fabricated.^102^ When CO_2_ is bubbled through the IL solution, it converts into a transparent, stable gel, which can be reverted to the initial solution state by purging with N_2_. Another study conducted by Tanaka et al. reported the simultaneous detection of hydrogen, ammonia and ethanol by an ionic gel with four electrodes.^103^ This study presented a multielectrode gas sensor comprising an [EMIM][BF4]-based ionic gel combined with Au, Cr, Pt and Rh electrodes. The gate voltage modulates the transistor drain current, which varies based on the electrode pairs and unintentionally absorbed gases. The voltage difference at the electrode or ionic gel interface changes the drain current in the transistor. For an effective sensor response, the electrode area must be larger than the gate area of the transistor, enabling potential downsizing and integration at the nanoscale. The bilayer interface plays a crucial role in gas detection, with chemisorbed hydrogen on Pt electrodes being the dominant factor in the sensor response.

By regulating intermolecular interactions through pH, changes in the pH environment can modify the optical, electrical, or mechanical properties of ion gels.^5^ Lin et al. fabricated a biocompatible hyperbranched poly(IL) containing gluconate (HPIL-Glu) to combat bacterial biofilms.^104^ The IL moieties consist of an ammonium-based cation and a Glu organic counter. The data indicated that HPIL-Glu forms a uniform nanoassembly in water and exhibits a pH-responsive charge conversion property. Under neutral conditions, Glu shields the positively charged surface, reducing toxicity. In a mildly acidic environment, Glu becomes protonated, exposing cationic groups that aid in the eradication of biofilms. Antimicrobial studies revealed that HPIL-Glu effectively kills bacteria and aids in healing bacterium-infected chronic wounds. In another study by Liu and colleagues, molecularly implanted poly(ILs) (MIPILSs) were imprinted onto multiwall carbon nanotubes to produce a pH-responsive surface by modifying MIPILs and bovine serum albumin (BSA) onto 3-aminopropyl triethoxysilane-modified MWCTs, resulting in a pH-responsive gel.^105^ Adsorption experiments revealed that the adsorption capacity of BSA by MWCNTs@BSA-MIPILs and MWCNTs@MIPILs decreases significantly with increasing pH from 7.7 to 9.9, with the imprinting factors increasing from 1.30 to 5.06. The optimal imprinting effect at pH 9.9 was due to weak electrostatic and hydrogen bonding interactions, which enhanced specific binding from the imprinted cavities. Santiago et al. fabricated spiropyran-based ionogel membranes using a florinated polymer, an IL and NO_2_BIPS spiropyran, which exhibited optimal thermos-, photo, halo-, and electrochromic switching behavior.^106^ The membranes are transparent, flexible and stretchable, exhibiting high ionic conductivity due to the plasticizing effect of the IL. NO_2_BIPS within IGs shows preserved photochromic, photohalochromic, thermochromic and electrochromic behavior, enabling reversible color changes under UV light, acid, heat and electric stimuli, respectively.

## 4 Self-Healing Mechanisms and Influential Aspects of Ionic Gels

A self-healing material is a substance that can autonomously repair and restore any damage it sustains. Self-healing ionic gels or polymeric gels can undergo self-repair in response to external triggers such as light, heat, or changes in pH. This repair process is facilitated by the interaction of covalent and noncovalent interactions between functional groups within the gel after it has been distorted or damaged.^52,107,108^ The self-healing properties of self-healing ionic gels or polymeric gels address their inherent limitations: they cannot recover spontaneously after being damaged. This advancement has significantly increased the potential of ionic gels and has expanded their applications in the field of biomedicine. This section summarizes and categorizes the design strategies of self-healing ion-based polymeric gels, which are based on dynamic covalent and noncovalent interactions, by considering the self-healing mechanism of these hydrogels.

### 4.1 Self-Healing Mechanism in Ionic Gels

**Figure 7:**
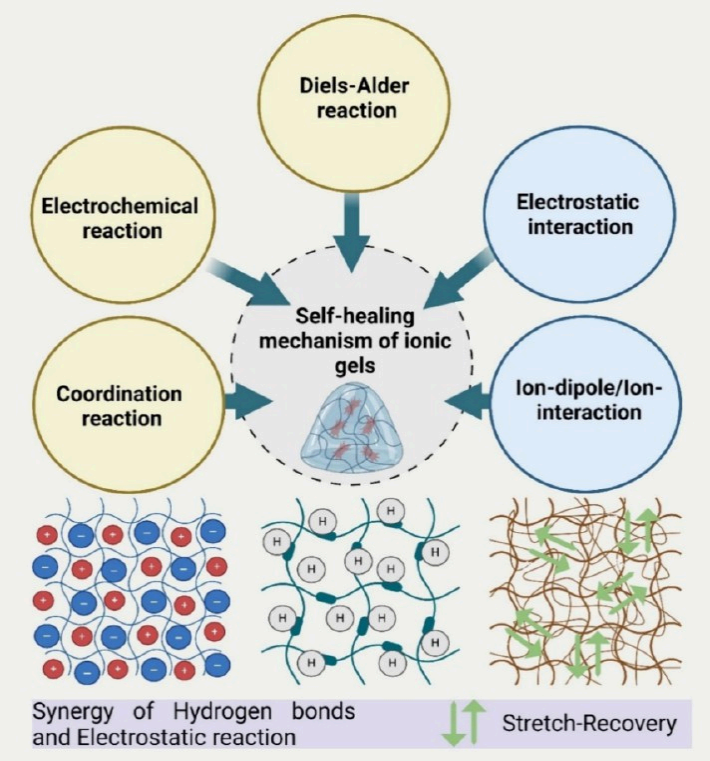
Schematic representation of the physical and chemical mechanisms involved in the formation of ionic gels. This image was created under a Creative Commons license using BioRender.

The self-healing mechanisms of ionic gels rely primarily on the reversible nature of their cross-linking structures, which frequently involve dynamic covalent bonds,^109,110^ such as Schiff base bonds, borate ester bonds,^111^ Diels–Alder reactions,^112^ disulfide bonds,^113^ and dynamic noncovalent bonding interactions, such as hydrogen bonding interactions,^110^ ion interactions (metal coordination),^114,115^ host–guest interactions,^116,117^ and hydrophobic interactions^118^ (Figure 7). The stability, self-healing ability, and mechanical qualities of ionic gels are directly influenced by the quantity and strength of the chemical bonds utilized in their production. Hence, researchers must understand the self-repair mechanism to devise ionogels with variable degrees of self-healing properties.

On a different note, the ions present in the ionic liquids also influence the gel’s properties to a greater extent. Owing to a greater number of anions and cations, ion–ion, ion–polar, and supramolecular contacts have greater strength than weak π–π interactions.^119^ Supramolecular ion interactions, specifically, exhibit exceptional stability and convenient accessibility, hence facilitating the development of self-healing materials.^120−123^ Simultaneously, the strong attraction between ion pairs promotes the reformation and unrestricted modification of polymeric chain structures. Thus, ionic liquids show great potential for integration into the production of self-healing ionic gels or composite gels. Ling et al. introduced physical doping of polydopamine nanoparticles (pDA-NPs) to enhance the mechanical characteristics and increase the pore size of a dynamic covalently cross-linked chitosan-arginine (CA) hydrogel via the use of a Schiff base.^124^ The administration of pDA-NPs successfully facilitated the healing process of significant skin injuries, augmented the formation of new blood vessels, and diminished the appearance of scars. In the context of tissue engineering, Lei et al. synthesized hydrogels by incorporating tannic acid (TA) and human-like collagen (HLC) into a dynamic cross-linking network of poly(vinyl alcohol) (PVA) and borax.^125^ In this system, borax acted as both a cross-linking agent and an ionic conductor. The incorporation of HLC and TA altered the cross-linking density and pH of the hydrogels, hence modifying the flexibility of the PVA-borax matrix and imparting hemostatic, antibacterial, anti-inflammatory, cell proliferative, and collagen deposition properties.

### 4.2 Factors Influencing Self-Healing Processes

Self-healing functional materials are influenced by several external and internal influences. Internal considerations include the potential for ionic transport within the material and the compatibility between ionic liquids and polymers. Conversely, certain intelligent self-repairing materials are sensitive to a range of external stimuli. Further investigations of these aspects may enhance our understanding of healing mechanisms and expand the range of potential applications.

#### 4.2.1 Ability to Diffuse Ions

The self-healing characteristics are significantly influenced by ion mobility. The utilization of hydrophobic interactions could be a viable approach for enhancing ion transport. To this end, Lee et al. developed polyampholyte hydrogels that exhibited high toughness, self-recovery and self-gealing characteristics that mimic those of human tissue.^126^ This was achieved by tuning the structure of the ion–pair associations, which act as cross-links in a polyampholyte terpolymer hydrogel with improved skin adhesion by adding a neutral monomer component to the network without changing the total charge balance. Variations in the neutral monomer feed concentration affected the network structure. In a different investigation by Sharma et al., an ionogel consisting of gelatin and 1-ethyl-3-methylimidazolium chloride was fabricated.^127^ When the ionogel was incised with a surgical blade, between 68% and 96% of the stiffness of the gel was restored at 20 °C within 10 h without the need for any external triggers. During the self-healing process, ion diffusion was driven by a hydrophobic contact between the alkyl tails of the ionic liquid molecules. Adequate chain mobility facilitates the active reorganization of ions accumulated on the fracture surface, resulting in the material possessing self-healing qualities that can enhance its mechanical and healing capabilities. Therefore, establishing the ideal equilibrium between superior mechanical strength and the dynamics of chains and ions is crucial.

#### 4.2.2 Interaction Compatibility of ILs with Polymeric Chains

The interaction between ILs and polymer chains has a significant effect on the self-healing capability and practical use of self-healing materials. To this end, a set of self-healing polymeric ionic liquid (PIL) hydrogels was created via hydrophobic association, resulting in hydrogels that possess both high mechanical strength and electrical conductivity.^128^ Hydrophilic monomer vinyl ionic liquids (VILs) derived from choline and amino acids, acrylamide, and the hydrophobic monomer stearyl methacrylate (C18) were combined in a micellar solution of sodium dodecyl sulfate (SDS) and polymerized together. Additionally, bacterial cellulose was added to improve the mechanical properties of the hydrogels. The hydrogels obtained displayed exceptional mechanical robustness, with a strength of 5.8 MPa, significant elongation at the point of rupture (4250%), and remarkable self-healing capability (85%) without the need for external intervention. Despite undergoing healing, the majority of the hydrogels achieved a tensile strength of only 2.5–3.9 MPa. Simultaneously, the integration of ionic liquids provided hydrogels with excellent electrical conductivity, reaching a maximum value of 1.258 S/m. Prasad et al. synthesized a form of guar gum (GG) by using 1-butyl-3-methyl imidazolium chloride [C4mim]Cl.^129^ In this solution, [C4mim]Cl served as both a solvent and a junction promoter, effectively connecting guar gum chains to create a network structure. Additionally, [C4mim]Cl facilitated the transformation of guar gum into a mechanical gel. The three-dimensional structure was reconstructed, resulting in the ability to self-heal itself. In a study reported by Zhu et al., a novel triple-network ion gel was synthesized using sustainable cellulose nanocrystals (CNCs) and ionic liquids instead.^130^ The gel exhibited greater mechanical strength and self-healing ability. By including graphene oxide and sustainable CNCs, the BmimHSO_4_-c ion gel showed enhanced mechanical properties, including high tensile strength (15.9 MPa), exceptional elongation (610%), and satisfactory toughness (53.8 kJ/m^3^). Furthermore, the presence of ions between Bmim^+^ in the ionic liquid and −OH in poly(vinyl alcohol) (PVA), along with hydrogen bonding inside the gel, led to a 93.8% increase in strength and a 93.3% increase in elongation after 24 h. Furthermore, the developed self-healing ion gel had notable and enduring self-healing capabilities.

#### 4.2.3 Self-Healing Initiated via Stimuli-Responsive Behavior

Self-healing gels or polymers can respond to many external stimuli to enhance their mechanical properties and healing capabilities. These stimuli include pH and temperature adjustments, electric current, and magnetic changes, among others.^131−134^ Alterations in environmental variables can expedite advancement and the ability to regenerate. In the case of thermally responsive self-healing materials, the enhancement of chain movement and diffusion, which is crucial for the healing process, can be achieved by increasing the temperature. To this end, Le et al. developed a bioinspired hydrogel depot that can be formed in the body and is sensitive to changes in pH and temperature.^135^ This hydrogel can be injected and used to manage the release of DNA-containing polyplexes. An array of multiblock copolymers, consisting of water-soluble poly(ethylene glycol) (PEG) and pH- and temperature-responsive poly(sulfamethazine ester urethane) (PSMEU), was used to create injectable hydrogels *in situ*. The PEG–PSMEU copolymer sols, which were in a free-flow state at high pH (pH 8.5) and room temperature (23 °C), were transformed into stable gels under physiological conditions (pH 7.4, 37 °C). A skin-mounted electrostimulation-augmented photothermal hydrogel patch (eT-patch) with a transparent ionic gel doped with MXene (Ti3C2Tx) was developed and applied to treat melanoma at 0.5 W/cm^2^.^136^ These ionic gels showed great photothermal conversion efficiency and electrical conductivity, improving the eT-patch. The exceptional optical transparency of the ionic gel-based eT patch allowed real-time skin reaction and melanoma treatment monitoring under photothermal and electrical stimulation. A systematic cellular study on the antitumor mechanism of eT patches under PES treatment revealed that they synergistically induce cancer cell apoptosis and pyroptosis, killing melanoma cells. Taken together, these findings suggest that owing to their safety and low side effects in healthy organs, eT patches are promising cost-effective treatments for skin tumors and could lead to new biomedical uses for ionic gels.

Magnetic gels are materials that possess both the magnetic properties of ferrofluids and the elastic qualities of hydrogels.^137^ For example, the magnetic–elastic connection enables the utilization of remote actuation by applying an external magnetic field.^138^ To this end, ferrofluid was developed by Hayashi and colleagues; it rapidly changes into a gel within tumors and produces heat when exposed to an alternating magnetic field (AMF).^139^ This ferrofluid was a result of magnetic substances (Fe_3_O_4_ nanoparticles), natural polysaccharides (alginate), and amino acids (cysteine). Notably, the fabricated gel also acted as a contrast agent for magnetic resonance imaging. The anticancer drug doxorubicin was loaded through the formation of hydrogen bonds. The application of AMF-induced thermal energy in gels made from ferrofluid-containing doxorubicin leads to the contraction of the gel and the release of doxorubicin. *In vivo* studies revealed that ferrofluid can be transformed into a gel specifically within the tumor and that this gel remains localized within the tumor.

## 5 Dermal Applications

Human skin can be replicated by a promising option as an ionic skin that is currently attracting significant interest in several applications.^140−142^ In general, a wide variety of hydrogels that can function as ionic skin are considered perfect because of their transparency, stretchability, and exceptional sensitivity. However, the limitations of conventional hydrogels, including poor environmental durability, can be overcome by ionic gels or ionogels that consist of a polymer network and several ionic liquids. Ionic liquids possess exceptional ionic conductivity and thermal stability, as well as significant contact with polymer chains.^143^ Self-healable materials are considered excellent for addressing the longevity of ionic skin, as they have the potential to repair mechanical damage. Several polymer materials with self-healing capabilities have been effectively engineered to improve durability and extend lifespan. The healing behavior can occur independently through the reversible nature of dynamic covalent connections.^133,144,145^

The growing need for biomaterials that can assist in the regeneration or replacement of injured tissue has led to the creation of novel engineered tissue structures in recent years. Nonetheless, ionic gels have been considered alternatives to these biomaterials in several biomedical applications, including contact lenses,^146^ wound dressings,^147^ and drug delivery vehicles,^148−150^ owing to their exceptional biocompatibility and biomimetic features. These self-healable ionic gels have been shown to possess specific characteristics that arise from their structure, which consists of a three-dimensional network of hydrophilic polymers that are highly swollen. This network can be chemically or physically cross-linked to create a material that imitates the beneficial properties of the well-hydrated extracellular matrix (ECM). Additionally, the porous structure of these gels enables the transport of nutrients and oxygen.^151^

### 5.1 Stimuli-Responsive Ionic Gels in Dermal Wound Healing

ILs have garnered significant interest because of their exceptional electrical conductivity, broad electrochemical range, and long-lasting nature. In the realm of biomedicine, exogenous electrical stimulation has proven to be a potent supplement to wound care. This is because the natural process of wound healing can be replicated by the use of internal electric fields to expedite the regeneration of skin.^152^ These self-healing qualities have great potential in the field of wound healing and protection. They can avoid secondary injuries, extend the lifespan of dressing materials, and provide additional protection for wound sites through various self-repair mechanisms. Kuddushi and colleagues created ester and salicylate-based ionic gels that possess the ability to repair themselves.^149^ These authors attributed this self-healing property to the dissociation and reforming of numerous hydrogen bonds between ionic liquids and polymers. A conductive and multifunctional hydrogel dressing was developed and reported by Liu et al.^153^ The dressing was fabricated using a combination of polymerized ionic liquid and konjac glucomannan. The hydrogel dressing showed exceptional mechanical qualities and biocompatibility. Additionally, it offered long-lasting and effective sterilization action without the need for the release of antibacterial substances. The utilization of this hydrogel dressing greatly increased the migration and proliferation of fibroblasts, as well as the therapeutic efficacy for diabetic skin wounds, by enhancing electrical stimulation.

**Figure 8:**
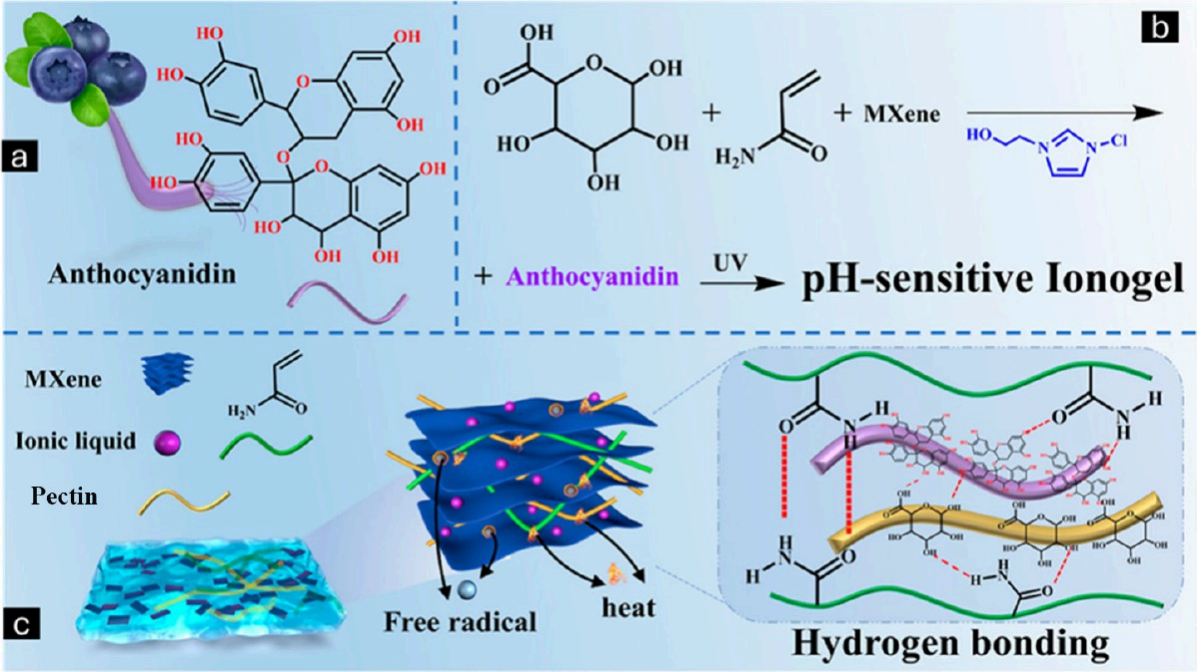
(a) Chemical structure of anthocyanidins. (b) Synthesis of the pH-sensitive ionogel. (c) Diagram illustrating the reaction dominated by hydrogen bonding. Adapted from ref (156). Copyright 2023 American Chemical Society.

Shou et al. developed HBCS-C hydrogels that are thermosensitive, injectable, tissue adhesive, biodegradable, biocompatible, and capable of promoting wound hemostasis.^154^ The liquid–gel transition displayed a high level of excellence at various temperatures because of variations in the hydrophilic–hydrophobic interactions and the formation of hydrogen bonds facilitated by the hydroxybutyl groups. Frequent interactions between tissues and catechol/amino groups enable biocompatible hydrogels to strongly adhere to tissue surfaces. In the study reported by Bhar et al., a wearable electroceutical platform (WEP) that produces weak electrical pulses was delivered to the wound site via a breathable electrical bandage patch.^155^ This patch was equipped with a silk-based antimicrobial ionogel interface. The evaluation of the *in vivo* efficacy of WEP revealed a notably accelerated wound-healing process. Histological and immunostaining examinations revealed that pulsed electrical stimulation (ES) led to an increased rate of granulation tissue creation, remodeling of the extracellular matrix, and regrowth of the epithelium. These modifications likely play a role in the reported improvements in the healing process. A transparent, pH-sensitive, extremely stretchable, and biocompatible anthocyanidin ionogel dressing was developed for precise and reliable green detection.^156^ The ionogel dressing demonstrated exceptional re-epithelialization during the 14-day wound healing process due to the antibacterial properties of the ionic liquid, the biocompatibility of the pectin, and the ability to eliminate free radicals from anthocyanidin. In addition, the pH values of the ionogel aligned with those of the usual wound exudate over 3 days were monitored. The resulting ionogel also exhibited favorable characteristics, such as effective water retention, swelling qualities, mechanical stretchability, and stability for 5 weeks. These findings highlight its significant potential for use in wound dressings (Figure 8).

**Figure 9:**
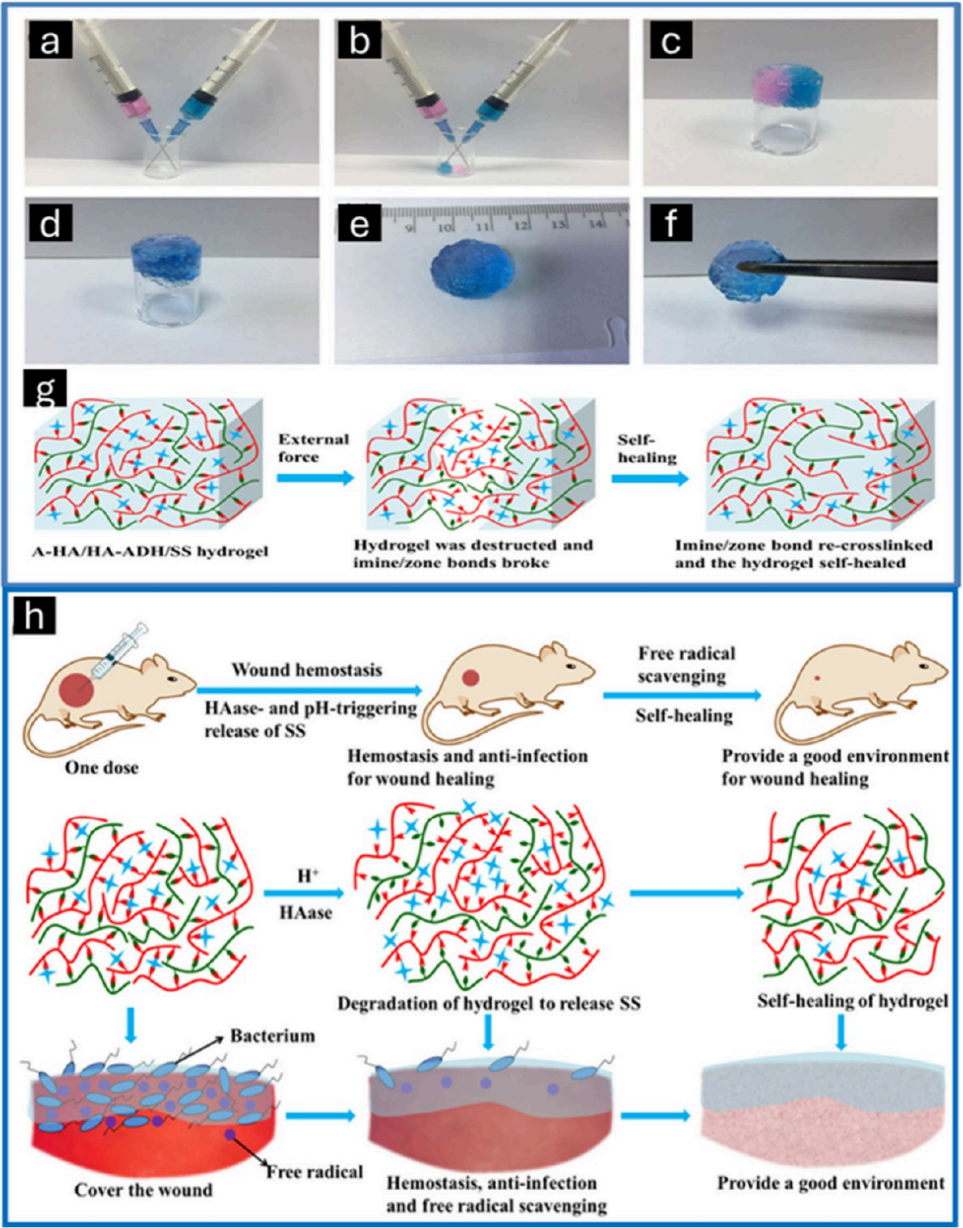
(a–f) Images of the ionic gel captured as an injectable and self-healing process at room temperature. (g) Schematic illustration of the self-healing mechanism of A-HA/HA-ADH/SS hydrogels. (h) HAase- and pH-sensitive A-HA/HA-ADH/SS hydrogels were developed to enhance wound healing by promoting hemostasis, preventing infections, scavenging free radicals, and facilitating self-healing. Adapted with permission from ref (166). Copyright 2020 Elsevier.

Stimuli-responsive ionic–polymeric hydrogel dressings have emerged as a highly promising approach for treating chronic and infected wounds. These dressings can release active biomolecules when needed to repair the local conditions in the wound area. The sol–gel transition of the hydrogel can be adjusted to provide the controlled release of growth factors that enhance the growth of epithelial cells while also providing a suitable structure for the development of the tissue matrix. Angiogenesis and collagen deposition can be expedited by incorporating the respective therapeutics into a stimuli-responsive polysaccharide hydrogel. In addition to ionic liquids, ionic-based polymeric gels, including chitosan, hyaluronic acid, and dextran, have been explored for the management of wound healing.^157^ These polymeric gels have shown the desired results. These polysaccharide-based hydrogels are responsive to stimuli, including pH, reactive oxygen species (ROS) and enzymes, and subsequently release drugs that can augment the typical bactericidal effect, modulate inflammation, and yield favorable outcomes in the wound healing process.^158^ Polysaccharides with aldehyde groups are often obtained by oxidizing natural polymers. These polysaccharides are routinely used to create hydrogels that contain acid-sensitive Schiff base cross-links. An example of such a polymer is oxidized hyaluronic acid (OHA), which is cross-linked with carboxymethyl chitosan to create a hydrogel used for diabetic wound treatment.^159^ In addition, aldehyde-functionalized synthetic polymers with complicated structures, specifically 4-arm poly(ethylene glycol) (PEG), have been cross-linked with natural polysaccharides such as carboxymethyl chitosan to treat chronic wounds.^160^ Recently, wound dressings that have both pH-responsive behavior and efficient antibacterial properties have become popular for managing inflammation and promoting wound healing. Hoque et al. introduced a method for creating a hydrogel with antibacterial properties and the ability to adhere to biological surfaces.^161^ The hydrogel was made by combining oxidized dextran with cationic quaternized chitosan. In addition, the hydrogels exhibited significant adhesive stresses ranging from 4.05 to 7.4 kPa, indicating strong bioadhesion. Wang and colleagues developed hydrogels that are responsive to changes in pH and temperature and can shield against UV radiation.^162^ These hydrogels were specifically designed to heal wounds in diabetic patients. The hydrogels were created by oxidizing the natural polymer pullulan to produce aldehyde pullulan, which was then cross-linked with Pluronic F127-grafted polyethylenimine by the creation of Schiff base bonds. Exosomes generated from adipose mesenchymal stem cells (ADSCs) are incorporated into hydrogels via electrostatic interactions.^163^ Enhanced release of the exosomes from the hydrogel was observed at pH 5.5 because of the breaking of the acid–labile imine bonds, which facilitated an efficient wound-healing process. Hydrogels that respond to both pH and reactive oxygen species (ROS) were reported by Hu et al.^164^ The hydrogel was made by modifying sodium alginate with 3-aminophenyl boronic acid, which allowed the boronic acid functions to be attached to the alginate backbone (ALG-BA). Hyaluronic acid (HA) is chemically modified by esterification with cholesterol (CHOL) molecules, leading to the formation of an amphiphilic HA-CHOL polymer. ALG-BA, when subjected to alkaline conditions with a pH range of 8–9, resulted in the formation of a hydrogel that contained boronic ester linkages that were sensitive to changes in pH and ROS. Zhao et al. created a photoresponsive hydrogel dressing with intelligent properties to increase the speed of wound healing.^165^ A supramolecular hydrogel was created through reversible interactions between azobenzene, and β-CD attached to hyaluronic acid chains. The flexibility of the hydrogel relies on the photoisomerization of azobenzene, which also has an affinity for the hydrophobic cavity of β-CD. When exposed to UV light, the connection between azobenzene and β-CD was altered, causing partial separation of the hydrogen bonds. Upon application, deliberate discharge of the enclosed EGF was accomplished as needed at the site of the wound. Dual stimuli-responsive hydrogels lead to an enhanced wound-healing process, as reported by Guan et al.^166^ The team developed a pH- and hyaluronidase-sensitive hydrogel cross-linked by OHA with HA-ADH. Histological analysis of a mouse skin defect model revealed a decrease in inflammation and pathogen count and a significant improvement in wound healing, with a 46% reduction in the wound area within 10 days (Figure 9). Furthermore, the hydrogel demonstrated the ability to self-heal with the application of an external force by regenerating its imine and acylhydrazone linkages. Wang et al. developed a hybrid gel for skin hemostasis.^167^ The gel consists of an interpenetrating network made up of pectin methacrylate and methacryloyl gelatin. Light and ions were used to produce cross-links in this gel. The high porosity of the gel network resulted in rapid absorption and coagulation in the blood. The properties of the gel were highly modifiable and readily detachable from the intended location. A pig skin bleeding model *in vitro* demonstrated that the ionic gel could be immediately injected into the wound and quickly photo-cross-linked, therefore reducing bleeding and shortening coagulation time by 39%. The cross-linked ionic gel can be effortlessly removed to avert additional wound damage. This injectable combination of PECMA/GelMA hydrogel was concluded to be a promising hemostatic agent. In a study by Kanaan et al., a semi-interpenetrating network (semi-IPN) of ionic gels composed of chitosan as electroresponsive biomaterials was developed.^168^ The objective of this study was to enhance and modify the electroresponsive and hemostatic properties of chitosan-based biomaterials. Poly(ionic liquids) or IL-based copolymers are produced by functionalizing the cations and/or anions of ILs with polymerizable chemical groups (such as vinyl groups) and then polymerizing and copolymerizing the resulting copolymers. The produced semi-IPN ionic gel showed excellent mechanical stability and was positively charged across a wide pH range, including basic pH. Under aqueous conditions at 32 °C, the semi-IPN ionic gel demonstrated enhanced release and penetration of lidocaine hydrochloride when subjected to an external electrical stimulus of 0.56 mA/cm^2^. The semi-IPN ionic gels were nonhemolytic (hemolytic index ≤0.2%) and showed high hemostatic activity (blood clotting index of ∼12 ± 1%).

### 5.2 Stimuli-Responsive Ionic Gels in Drug Delivery

#### 5.2.1 Stimuli-Responsive Ionogels in Chemotherapeutic Delivery

In cancer therapy, diverse nanocarriers such as liposomes, micelles, dendrimers, and metal nanoparticles are employed to decrease necessary treatment dosages and alleviate systemic off-target side effects associated with oral or parental administration. Nonetheless, these carriers exhibit constraints including cyclic instability and inadequate drug solubility, among others.^169^ The topical application of gels loaded with ionic components is beneficial to circumvent these limitations. Stimuli-responsive ionic gels are recognized for modifying physicochemical characteristics such as color, transparency, conductivity, and swelling in reaction to stimuli, providing great potential as carriers for targeted chemotherapy drug delivery. The ongoing studies have shown that ILs may possess anticancer effects by triggering cytotoxicity in cancer cells. They have undergone testing on multiple cancer cell lines, including those for breast cancer, melanoma, and liver cancer. However, the precise mechanism by which ILs demonstrate their anticancer properties remains under investigation. Certain studies indicate that they may induce DNA damage, affect cellular membranes, or interfere with cellular energy metabolism.^170,171^

Kuddushi et al. reported a low molecular weight ionic liquid gelator, cetylpyridinium salicylate (CetPySal), that formed an ionogel at a critical gelation concentration of 4.70% w/v.^172^ The ionogel demonstrated a temperature-responsive phase transition from opaque to transparent due to structural changes in both phases. The phase behavior was analyzed using several advanced analytical approaches. The dilution stability of the hybrid pharmaceutical ionogel was achieved by encapsulating the anticancer drug, imatinib mesylate (IM) within the ionogel matrix. *In vitro* release studies illustrated the release profile of IM from the ionogel matrix in a release medium at pH 10, indicative of a basic environment, pH 5, representative of the acidic milieu of extracellular tumor tissues, and pH 7.4, reflecting the pH of normal tissues. A notable pH-dependent behavior was found at 37 °C. IM exhibited a gradual release at pH 10 (53.17%) and pH 7.4 (88.3%) from the ionogel matrix. Approximately 53.17% and 88.3% of the IM was released in 330 min at pH levels of 10 and 7.4, respectively. The release rate of IM was significantly improved in acidic conditions. IM exhibited a release of 94.17% within 210 min at pH 5. Overall, the release data indicated that drug release from the ionogel matrix primarily occurred via surface erosion The alteration in surface area and dimensions of the micellar aggregates resulting from temperature variations also influenced the release kinetics. These data were anticipated to be beneficial for utilizing the API-based IL, CetPySal, in chemotherapeutic delivery applications.

In another study, the same group reported a range of functionalized ionic liquid-based gels specifically designed to deliver chemotherapeutic drug, DOX.^173^ The team fabricated a polymeric ionic hydrogel using surfactants based on ionic liquids (CnEMorph)Br, which were polymerized with poly(vinyl) alcohol. This hydrogel was designed to respond to stimuli and enable the controlled release of DOX. Approximately 82.3% of the DOX diffused within 50 h at 37 °C and a pH of 5.0. An ionic hydrogel for cancer treatment was developed by employing a salicylate-based active pharmaceutical ingredient without the inclusion of any other additives or cross-linking agents.^174−176^ Under mildly acidic conditions, there was a heightened release of DOX, possibly attributed to the protonation of the hydrogel. Kuddushi et al. developed a hydrogel composed of an API-based IL that possessed self-healing and injectable characteristics. The ionic hydrogel serves as a localized, long-term codelivery system characterized by self-healing, injectability, and stimuli-responsive attributes.^177^ The smart hybrid ionic hydrogel was developed by encapsulating DOX within a three-dimensional matrix that responds to intracellular biological cues, such as pH and temperature. At 37 °C and pH 5.0, the percentage of DOX release from the ionic hydrogel was effectively regulated and persisted for over 57 h. No rapid DOX release was detected in the early phase, however, the percentage of release exceeded around 86.4% in the subsequent phase. The release percentage was around 45.3% at pH 7.4 for a duration of 57 h. Analogous findings were noted when the hydrogel was incubated at 25 °C. The total DOX release was approximately 33.2% at pH 5.0 and approximately 18.2% at pH 7.4. The acidic pH enhanced the release of DOX, due to the increased hydrophilicity of DOX and its desolubilization inside the ionic hydrogel fibrous network. In conclusion, these intelligent ionic hydrogels exhibited a release of DOX regulated by dual stimuli: temperature and pH. *In vitro* cellular investigations have shown that the DOX-loaded hybrid ionic hydrogel exhibits a synergistic anticancer impact against MCF-07 cells. A stimuli-responsive, self-healing, sticky, and injectable polymeric hydrogel including an ester-functionalized ionic liquid as an adjuvant to enhance the encapsulation and localized delivery efficiency of DOX was reported.^178^ The engineered polymeric hydrogel reacted to intracellular biological stimuli (e.g., acidic pH of cancerous cells and temperature), altered its morphology by modifying the shape and size of the gelator within the hydrogel matrix, and efficiently released DOX at the tumor site. The *in vitro* release studies showed a cumulative release of 82.3% DOX at 37 °C and pH 5.0 after 50 h, compared to 53% at pH 7.4. When incubated at 25 °C, the polymeric hydrogel showed a similar pattern. The cumulative DOX release was ∼64.0% at pH 5.0 and ∼22.3% at pH 7.4. The *in vitro* cytotoxicity and drug release analysis demonstrated that the hybrid hydrogel is more efficacious in eliminating malignant cells, with the tailored release of DOX occurring at intracellular acidic pH levels.

**Figure 10:**
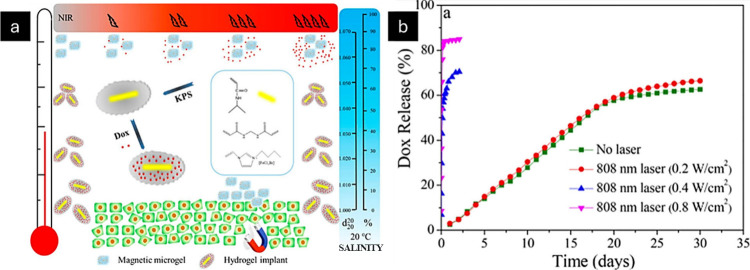
(a) Schematic representation of magnetic hydrogels for near-infrared modulated on-demand cancer therapy. (b) DOX release patterns from hydrogel implants over 30 days, with or without near-infrared laser application at varying power densities. Adapted from ref (181). Copyright 2019 American Chemical Society.

A thermoresponsive nanogel made from poly(lactide)-*g*-pullulan (PLP1 and 2) copolymers, which have a phase transition temperature of 35 °C, have been reported as carriers for delivering DOX.^179^ A cytotoxicity assay conducted on HeLa cells revealed that the IC_50_ values of DOX released from PLP 1 were approximately 5.9 and 9.3 mg/mL at 37 and 42 °C, respectively. This indicated a 1.6-fold increase in cytotoxicity at 42 °C compared with that at 37 °C, which was attributed to the greater release of DOX at this temperature. Yuan et al. described the synthesis of biocompatible and biodegradable nanogels with a branching structure.^180^ This was achieved by reacting 3,6-dioxaoctan-1,8-diyl bis(ethylene phosphate) (TEGDP) with tris (2-aminoethyl)amine (TREN) in an ionic liquid that contained a miniemulsion. The nanogels effectively encapsulated DOX, exhibiting an enzyme-triggered drug release mechanism, and were successfully internalized by the human breast cancer cell line MDA-MB-231. Wang et al. reported a novel methodology for the fabrication of pharmacological implants using hydrogels, leveraging the collective behavior of jagged magnetic microgels, which are enhanced by covering Au nanorod@SiO_2_ with thermo- and magnetic-responsive polymer shells composed of poly(*N*-isopropylacrylamide-*co*-magnetic ionic liquids).^181^ The magnetism of subsequent macroscale hydrogels was augmented roughly 5-fold due to the self-organization process, providing new proof for the fundamental nature of magnetism creation at the molecular level. Utilizing near-IR laser excitation, these hydrogel implants exhibit little cytotoxicity and good biocompatibility, enabling them to serve as local drug delivery systems for sustained release of DOX over 30 days, while also facilitating on-demand release for improved therapeutic efficacy (Figure 10). In a nutshell, the fabricated microgel colloids offered a novel approach to reorganizing molecular magnets and presented a distinct opportunity for the increase of magnetism. This enhancement promoted the advancement of solid tumor therapy and provided an impetus for the actual implementation of on-demand medication treatment.

Parsana et al. developed a self-healable, injectable, and ionic conductive supramolecular eutectogel utilizing a natural deep eutectic solvent (NADES) loaded with curcumin, an anticancer drug.^182^ The investigated eutectogels were synthesized by dissolving the pharmacologically active cetylpyridinium chloride (C_16_PyCl) and cetylpyridinium bromide (C_16_PyBr) in the NADES. The NADES was created by the interaction of choline chloride (ChCl) with mono-, di-, and trimeric acids, specifically formic acid (FA), oxalic acid (OA), and citric acid (CA) via hydrogen bonding. The eutectogel exhibited superior self-healing, injectability, and ionic conductivity, alongside remarkable antibacterial characteristics and great encapsulation efficiency for curcumin. The sustained release properties and release kinetics of curcumin were also examined. The dialysis method was employed to facilitate *in vitro* curcumin release under physiological circumstances (pH 7.4 and 37 °C). During the initial hour, 20% of the curcumin was released; roughly 50% was released in the second hour, and 97% of the total curcumin loaded in the COPBr gel was released in the third hour. In the 3CCPBr gel, 22% of the total curcumin was released in the first hour, 51% in the second hour, and approximately 98% in the third hour. In summary, the development of a novel supramolecular eutectogel can be used for prolonged drug release for smart drug delivery systems. A novel advancement was made by producing a hybrid ionic hydrogel using choline oleate-IL and PVA.^183^ This gel has shown a remarkable ability to efficiently encapsulate curcumin, an active component with anticancer and antibacterial properties, without any degradation. The *in vitro* biocompatibility of the hydrogel on normal human L-132 cells was examined, revealing a cell viability of 92% after 48 h. The curcumin-loaded formulation was subsequently examined for its sustained release in phosphate-buffered saline (PBS) at pH 7.4 and pH 5.0, at temperatures of 25 and 37 °C, respectively, during specified time intervals ranging from 0 to 75 h. The results showed a cumulative release of 85% of curcumin after 75 h at 37 °C and pH 5.0, in contrast to a 69% release at pH 7.4 over the same duration. Curcumin was released at 25 °C, with 74% at pH 5.0 and 49% at pH 7.4 during the same duration. Overall, the prolonged release of the multifunctional hydrogel rendered it a significant application in the domains of anticancer therapeutic delivery.

In a different investigation, Shayanfar et al. developed an ionic gel system loaded with sunitinib malate (SUM), an anticancer drug.^184^ The drug-loaded ionic gel was synthesized by polymerizing 2-hydroxyethyl methacrylate in a mixture containing SUM and choline chloride/ascorbic acid. The ionic gel demonstrated biocompatibility, creating a favorable environment for cells. The study revealed that the drug was released at a higher rate from the ionic gel system at pH 1.2 than at pH 6.8 and pH 7.2. This was attributed to the amino groups on SUM becoming protonated. The impact of pH on gel formation was effective within the pH range of 1.7--3.5.

In a study by Shekaari et al., the potential mechanism for the development of ion-gels utilizing therapeutic deep eutectic solvents (THDESs) for drug delivery systems, specifically examining their interaction with the anticancer drug 5-fluorouracil (5-FU) was explored.^185^ This study consisted of two phases: initially, a THDES comprising choline chloride (ChCl) and ascorbic acid (AA) was synthesized, followed by the acquisition of certain thermophysical parameters of the THDES using density and sound speed measurements at varying temperatures. The second phase involved the production, characterization, interaction analysis, and drug release profile of a novel biodegradable, eco-friendly, and sustainable carrier for 5-FU, incorporating a THDES [ChCl)/AA] and 2-hydroxyethyl methacrylate (HEMA). The drug release patterns of the ion-gel were assessed in two distinct pH levels (6.8 and 7.4). The results indicated that this novel drug delivery mechanism for 5-FU as an anticancer agent may occur naturally, with ion-gel release of 5-FU at around 68% and 39% under pH values of 6.8 and 7.4, respectively. A strategic nanoparticle-free method for creating magnetoresponsive biocomposite hydrogels by integrating a biopolymer (gelatin) with vesicles of an essential amino acid (valine)-based magnetic ionic liquid surfactant [ValC_16_][FeCl_4_], was investigated as drug delivery nanocarriers.^186^ The fabricated biocomposite hydrogel has been utilized as a delivery carrier for an antibiotic (ornidazole) and an anticancer agent (5-FU). The encapsulation efficiencies of ornidazole and 5-FU in the magnetic biocomposite gel were determined to be 69 ± 0.6% and 78 ± 0.3%, respectively. Overall, magnetoresponsive biomaterials may be spatially and temporally altered using an external magnetic field, making the developed nanoparticle-free magnetoresponsive hydrogels potential candidates for active scaffolds in improved drug delivery and tissue regeneration applications.

Taken together, the development of innovative ways for successful cancer treatments, aimed at enhancing therapeutic outcomes and minimizing systemic toxicity linked to conventional medications, is critically important. Stimuli-responsive ionogels have enabled the development of sustained and precise targeted drug delivery systems for anticancer agents at the tumor site. The release of drugs at tumor sites can be adjusted by using the unique physiological properties of the tumor microenvironment, specifically the varying extracellular and intracellular pH levels. Furthermore, the utilization of light, magnetic fields, or modifications in ionic strength can control the pace and site of drug release. It is anticipated that, shortly, novel smart materials and formulations developed and validated for this purpose will likely enter clinical trials, with a translational approach.

#### 5.2.2 Stimuli-Responsive Ionic Gels for Multifaceted Disease Management

Dermal drug delivery systems are attractive routes for the delivery of small- to high-molecular-weight drugs. Drug delivery is dependent on the physicochemical properties of the therapeutic biomolecules and the structural arrangement of the skin. It is believed that the stratum corneum, the outermost layer of the skin, is a formidable barrier to drug permeation. To this end, various physical and chemical techniques have been implemented and have been proven to enhance the delivery of therapeutics across the stratum corneum for retention within the viable epidermis or into the systemic circulation to achieve the desired therapeutic effects.^187^ Mukesh and Prasad developed a pH-responsive DNA-based soft ion gel by utilizing a deep eutectic solvent mixture of choline chloride and ethylene glycol (ChCl-EG 1:2).^188^ The gel exhibited breakdown under alkaline conditions at a pH of 7.3 and then underwent structural reconstruction under acidic conditions at a pH of 2.9, thereby paving a pathway for its applicability in slow and targeted drug/gene delivery systems, as well as in other advanced applications requiring such DNA structures. Ionic liquid-based gel systems have shown significant results from the studies that explored dermal drug delivery for antibacterial, anti-inflammatory, and antiepileptic, among others (Table 1).

**Table 1:** Summary of Applications of Drug-Loaded Stimuli-Responsive Ionic Gels in Different Disease Treatments^a^

stimulus	therapeutics	ionic components	disease	outcome	ref
Temperature	DS and IM	C_12_EMeImBr, C_14_EMeImBr, C_16_EMeImBr, C_12_VnImBr, C_14_VnImBr, C_16_VnImBr, C_12_MeImBr, C_14_MeImBr, C_16_MeImBr, C_12_TABr, C_14_TABr, C_16_TABr	Wound	Demonstrated excellent dye-absorbing and drug-encapsulating properties	(189)
Temperature	CUR	Ester functionalized ionic liquid, 3-methyl-1-(hexadecyloxycarbonylmethyl)imidazolium bromide (C_16_EMeImBr)	Wound	Exhibits excellent metal ion absorption properties and showed controlled release with enhanced stability	(190)
Temperature	DS	Choline acetate-IL	Inflammation	Tunable elastic and viscous behavior, with modified drug release	(191)
Temperature	PB	Choline acetate, choline dihydrogen phosphate, and choline chloride	Epilepsy	The ionogel exhibited self-healing characteristics, tunable mechanical and structural properties, high thermal stability, electroconductivity and improved solubility and stability of the drug	(61)
pH and glucose response	–	Pyrrolidinium ILs	Bacterial infections	Exhibited enhanced antibacterial activity, having potential for effective joint wound dressing due to their stretchability and adhesive properties	(46)
Microwave stimulated	Levo	Vinylbenzyl trimethylammonium chloride and [2-(methacryloyloxy)ethyl] trimethylammonium chloride	Bacterial and deep tissue infections	Enhanced thermal conversion and effectively killed *S. aureus* and MRSA when triggered by microwave	(192)
Temperature	MTX	CAGE-IL	Psoriasis	Enhanced the penetration of MTX (27.6%). *In vivo* experiments demonstrated that the MTX/ME-Gel formulation was able to alleviate skin redness, swelling, and scaling induced by imiquimod.	(193)

a Abbreviations: DS, diclofenac sodium; IM, imatinib mesylate; CUR, curcumin; PB, phenobarbital; Levo, levofloxacin; MTX, methotrexate.

## 6 Tissue Engineering Applications

The tissue engineering field addresses regenerating and revitalizing damaged tissues or organs via scaffolds made of various biodegradable and biomimetic materials. Scaffolds are three-dimensional solid structures that provide a physical framework to cells, allowing them to colonize and differentiate into functional tissue by immobilizing proteins and growth factors. Scaffolds made of hydrogels have gained research focus because of their ability to organize various cells or 3D models by mimicking the extracellular matrix present in the body (Table 2). Designing a scaffold that modifies its physical properties depending on stimuli is a smart approach. Stimuli might originate from either external or internal sources. External stimuli refer to stimuli that are generated outside the body. Light, electricity, and magnetism are examples of external stimuli. Internal stimuli refer to the temperature and enzymes that arise within the body due to physiological or pathological conditions. Temperature-sensitive hydrogels are a widely studied class of hydrogel systems formed from polymer solutions with relatively low critical solution temperatures. They exhibit inverse temperature dependencies and are characterized by hydrophobic and hydrophilic segments. Modifying naturally available polymers or combining them with synthetic polymers offers new hydrogel development possibilities.

**Table 2:** Various Designs of Stimuli-Sensitive Hydrogel Scaffolds for Tissue Engineering^a^

stimulus	polymer	type of cells	gelling technique	applications	ref(s)
Temperature	Chitosan-β glycerophosphate	bone marrow stem cells	freeze-drying	bone tissue engineering	(260)
	pNIPAAM–Gelatin	cardiac fibroblasts and cardiomyocytes	free radical polymerization	cardiac cells delivery	(275)
	Elastin-like polypeptides with polyaspartic acid	umbilical vein endothelial cells	sol–gel transition	provides a 3D microenvironment and controls cellular functions	(262)
	pNIPAAM-chitosan	vascular endothelial cells	free radical polymerization	promotes angiogenesis	(261)
Light	GelMA	primary cardiomyocytes	UV irradiation	myocardium healing and treating cardiovascular diseases	(276)
		odontoblast-like cells	UV irradiation	regenerative dentistry	(264)
	Polyamidoamine	endothelial cells	free-radical polymerization	*in vitro* model for drug screening	(277)
	Poly(ethylene glycol) diacrylate	mesenchymal stem cells	UV irradiation	bioresponsive hydrogel for tissue engineering	(265)
	Methacrylate poly(ethylene glycol-*co*-depsipeptide)	endothelial cells	stereolithography	tissue engineering	(266)
Electric	Poly(acrylic acid)-fibrin	Smooth muscle cells	free radical polymerization	To generate tissue-engineered blood vessels	(270)
	GelMA-graphene oxide	rat pheochromocytoma cell line	UV irradiation	Neural tissue regeneration	(268), (269)
	GelMa-polypyrrole-oxidized HA	Neuronal cells	UV irradiation	Dental implants integration	(278)
Magnetic	Type ii collagen-HA-magnetic nanoparticles	Mesenchymal stem cells	chemical cross-linking	Scaffold for cartilage tissue engineering	(279)
	Xanthan gum-chitosan with iron oxide nanoparticles	NIH3T3 cells	physical cross-linking	Skin, muscle, cartilage and connective tissue engineering	(272)
	Gelatin with magnetic nanoparticles	stem cells	enzymatic cross-linking	Anisotropic tissue engineering	(280)
	Poly(ethylene glycol) diacrylate–SPION	mesenchymal stem cells	thermal radical polymerization	Tissue engineering in deeper tissues	(271)
Biochemical	Poly(ethylene glycol)–peptide	Mesenchymal stem cells	chemical cross-linking	Tissue engineering using biomaterial carriers	(273)
		Chondrocytes	UV irradiation	Cartilage development	(281)
	Gelatin	Fibroblasts and/or endothelial cells with macrophages	enzymatic cross-linking	Immune cells or cytokines-based tissue engineering	(274)

a GelMa, gelatin methacrylate; HA, hyaluronic acid; SPION, superparamagnetic iron oxide nanoparticles.

### 6.1 Scaffold Design and Fabrication

By offering a supporting framework for the growth and regeneration of injured tissue, hydrogel scaffolds play crucial roles in tissue engineering. These scaffolds are created via several fabrication processes, each with benefits and difficulties. These methods are crucial for creating efficient frameworks for tissue regeneration purposes. Some commonly used methods for manufacturing are electrospinning, 3D bioprinting, and freeze-drying. These techniques have distinct advantages, such as the ability to manipulate the structure and porosity of the scaffold precisely.

#### 6.1.1 Electrospinning

Electrospinning is a long-standing and cost-effective technique for producing submicron-sized fibers with well-formed mesh structures. The system employs a syringe pump, a high-voltage direct current source, and a grounded revolving collector. An electric current is initially sent through the syringe tube containing the polymeric material. This causes electric repulsion inside the polymer solution, resulting in the ejection of the polymer from the nozzle tip as thin filamentous strands. The fibers are gathered by the revolving target collector based on the desired characteristics of the scaffold.^194^ The size attained with this technique is often smaller than that obtained via alternative methods. The scaffold exhibited excellent cell contact, characterized by robust proliferation, strong adhesion, and effective differentiation.^195,196^

#### 6.1.2 Photolithography

In recent years, the photolithography approach has been extensively employed to create polymeric 3D scaffolds that are based on selective lighting methods.^197^ The construction adheres to a biphasic approach. The photoresponsive polymer is first covered in a mask with appropriate shapes and sizes over the substrate to enable photopolymerization in the exposed regions. This mixture is then exposed to UV light. Next, the remaining unreacted and unexposed polymeric substrate is removed via a solvent, forming patterned 3D scaffolds.^198−200^ Owing to its very specialized manufacturing method, photolithography may be used to preserve great pattern architecture and alignment while producing 3D patterns on larger surface areas.

#### 6.1.3 Freeze-Drying

The freeze-drying approach involves adding a polymeric component and a solvent to water to create an emulsion. This solution is subjected to fast cooling, reaching temperatures between −70 °C and −80 °C, resulting in thermal instability within its structure. In addition, this condensed substance in a reduced-pressure environment undergoes sublimation, causing the solvent to vaporize and ultimately create empty spaces in the freeze-dried structure, constructing the scaffolds.^201^ The lyophilization process helps regulate the pore size and thus the permeability of the scaffolds. Multiple studies on this manufacturing technique have shown its capacity to preserve a porous structure.^202^

#### 6.1.4 3D Printing

Modern 3D printing technologies have surpassed traditional methods for making hydrogels. These advanced techniques may create hydrogels with specific sizes, forms, and structures and diverse scaffold systems with specific functions. 3D printing enables precise manipulation of biological structures for transplantation, allowing accurate control over geometry and cell distribution inside the scaffold.^203^ This technology allows the creation of 3D designs that closely replicate the donated organ. This approach uses computer-aided design to create a model of the tissue that will be transplanted. The model is then produced quickly via rapid prototyping methods. The ink droplets build a layer-by-layer framework, creating an intricate tissue architecture suitable for implantation.^204,205^ Nevertheless, inkjet/3D printing is restricted in terms of resolution, raw materials, and the expense associated with manufacturing. Further research is necessary to enhance the development of inks containing biological components specifically intended for tissue engineering applications.^206^

## 7 Cell Encapsulation and Microencapsulation Techniques

In 1964, Chang invented the notion of encapsulation, which involved enclosing enzymes in a thin polymer membrane in an attempt to introduce them into the body.^207^ After 20 years, the experiments incorporated islet cells into the body via a polyethylenimine membrane.^208^ Tissue engineering involves the transplantation of cells to a damaged area to develop functional tissue over time. Nevertheless, the transplanted cells may be recognized as foreign entities and subsequently ingested by the innate immune system. Thus, employing a polymer matrix to enclose cells to safeguard them while still allowing for the transport of oxygen and nutrients would be the most secure method.^209,210^ Cell encapsulation can be achieved via either macro- or micro techniques.

**Figure 11:**
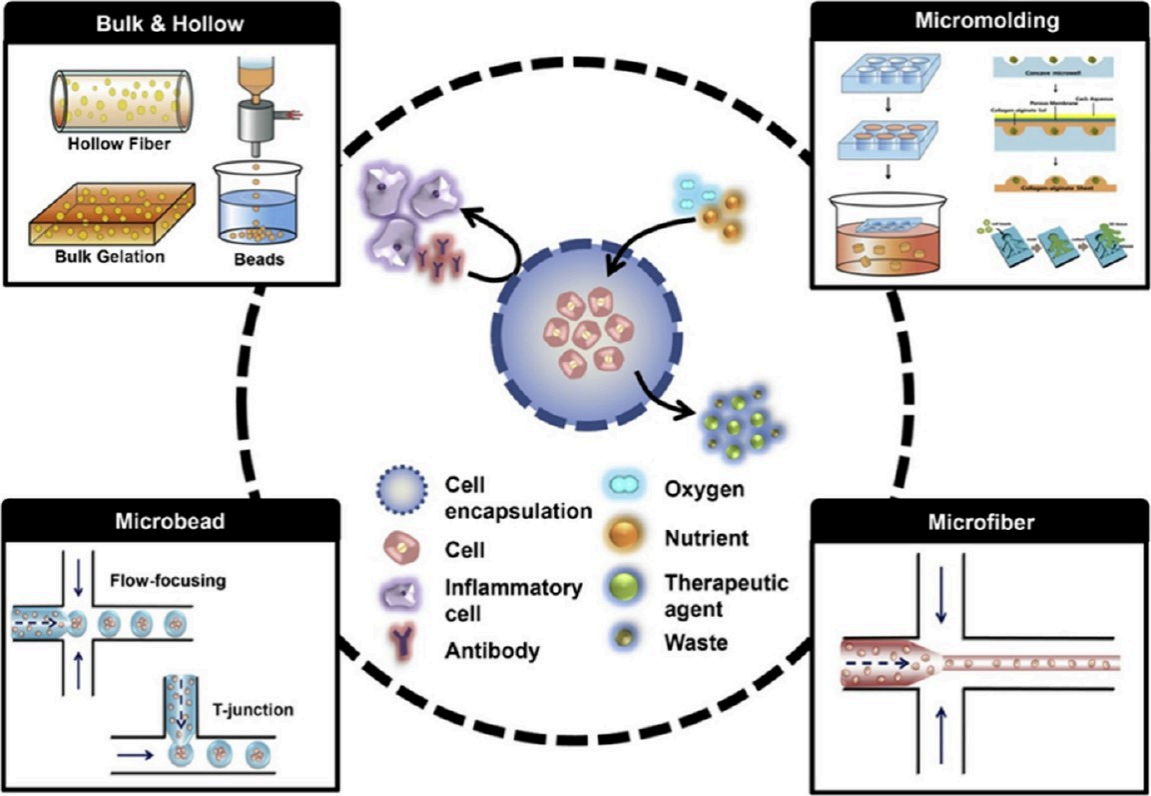
Schematic representation of different cell encapsulation techniques. Adapted with permission from ref (222). Copyright 2014 Elsevier.

Microencapsulation procedures involve the suspension of cells in a hydrogel that is in a pregel condition. Cross-linking is then initiated through a chemical reaction, a temperature change, or exposure to light. Polyethylene glycol (PEG) is the most extensively researched synthetic polymer for macro molding and is often accompanied by cross-linkers and gelation initiators. Typically, PEG-based hydrogels rely on photopolymerization for cross-linking.^211−213^ However, Amit and his team successfully created a polymer called poly(ethylene glycol) (PEG)–poly(*N*-isopropylacrylamide) (PNIPAAM), which forms cross-links when exposed to changes in temperature.^214^ Macromolding encapsulation is a relatively simple approach that is utilized for various types of cell lines, such as pancreatic cells,^215−217^ neuronal cells,^218^ endothelial cells,^219^ smooth muscle cells^220^ and hepatocytes.^221^ Nevertheless, the main difficulties lie in the necrotic zone located at the core of the scaffolds and the vulnerable encapsulating membranes that are prone to tearing when subjected to mechanical abrasion.^222^Figure 11 shows a schematic representation of macro- and microencapsulation techniques.

Microencapsulation techniques were created to address the constraints of macro methods. Microscopic hydrogels efficiently encapsulate a standardized number of cells, improving the uniformity and suitability of the approach in a clinical environment. Electronics and hydrodynamics are employed to facilitate the application of electrospraying, micromolding, and microfluidics. The electrospray technique is the most commonly investigated method for microencapsulation. This involves spraying a polymer solution containing cells via a voltage gradient to create homogeneous microdroplets. This approach has also been used to create stimuli-responsive hydrogels as tissue-regenerating scaffolds and drug delivery systems for treating chronic wounds and cancer.^223,224^ The quality of cell encapsulation is significantly influenced by critical process factors such as the nozzle diameter, flow rate, and voltage. To obtain the ideal droplet size and cell density, it is imperative to optimize the parameters discussed above. Using a moderate voltage to ensure the highest possible cell viability is particularly advantageous. The electrospray method has been used to explore polymers such as alginate, gelatin, and polycaprolactone extensively. Microbeads containing mesenchymal stem cells, which were placed in a gelatin matrix sensitive to temperature, showed a beneficial effect on wound healing in a live model after 7 days of treatment.^224^ A study on cartilage regeneration revealed that the combination of alginate and gelatin was more effective in encouraging the proliferation of human bone marrow stem cells than was alginate alone.^225^

Micromolding is a method that often utilizes polymers that are responsive to light or temperature. This technology enables the production of hydrogels of various shapes by filling molds with a polymer solution containing suspended cells. A study was conducted to investigate the use of fibrin in developing scaffolds with precise architecture through photopolymerization. This work revealed that the micromolding approach may be employed to create 3D structures from proteins.^226^ Similarly, hyaluronic acid, a type of polysaccharide, is chemically altered such that it becomes sensitive to light and then combines with fibroblasts to create a matrix that is compatible with living organisms.^227^ Unlike photolithargic-based micromolding, the Ma group created a method using pneumatics to produce precise geometric microgels that can accommodate a wide range of cells. The creation of a multicompartment microgel enabled the formation of 3D liver organoids with hepatic cords enveloping them.^228^

**Figure 12:**
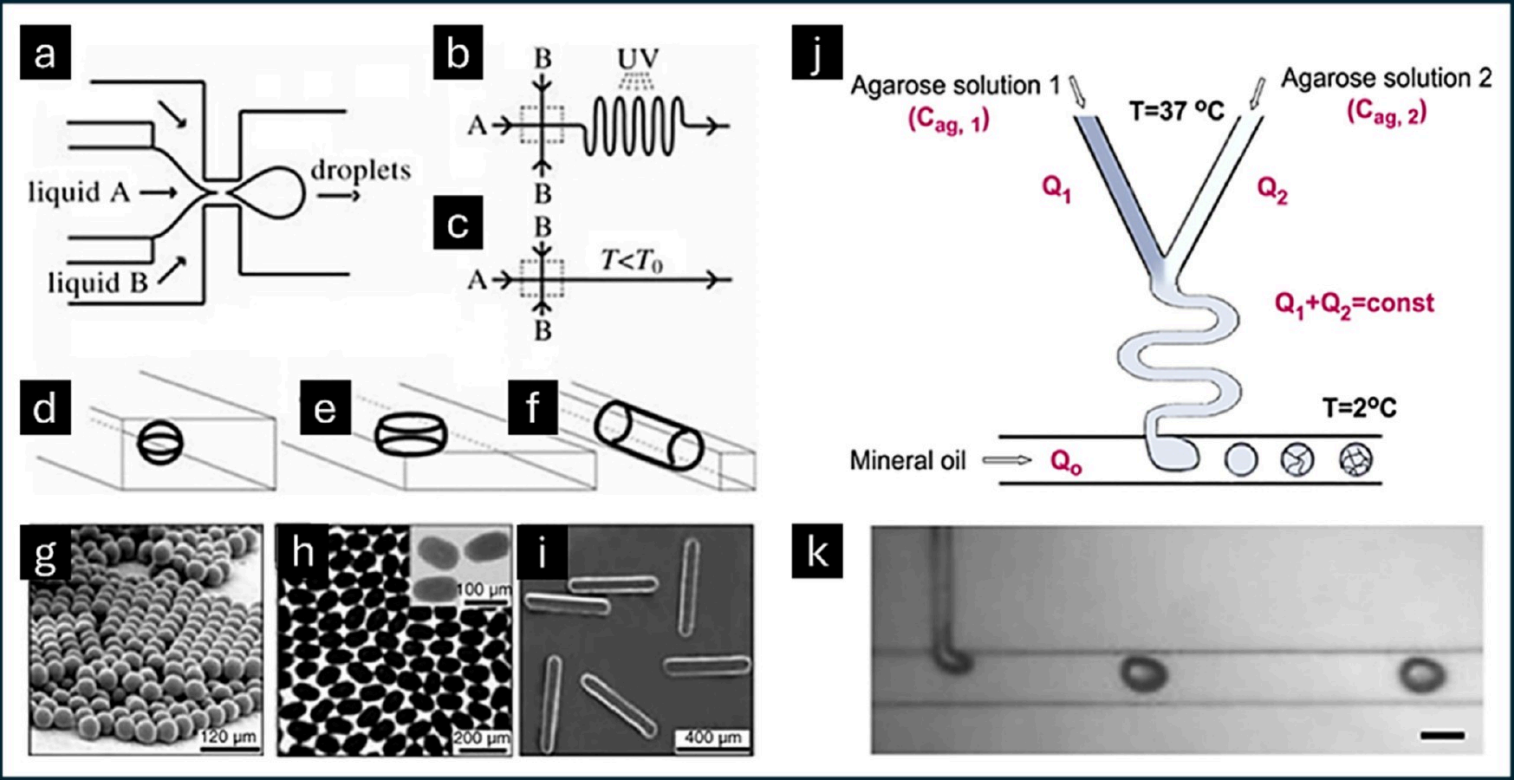
(a) Depiction of the flow-focusing configuration employed in the microfluidic droplet generator. The two immiscible liquids A and B are compelled through the tight aperture, causing the inner liquid core to rupture and release monodisperse droplets into the exit channel. (b, c) Illustrations of the apparatus employed for the generation of photochemically and thermally solidified particles. The channels utilized for photochemical cross-linking were extended to provide prolonged exposure of the droplets to UV light. In thermal setup tests, the flow-focusing area was maintained at a temperature above the gelling (or solid–liquid phase transition) temperature (*T*_0_). The outlet channel was chilled to a temperature below *T*_0_, causing the droplets to solidify as they traversed the channel. (d–f) Illustrations of the configurations of droplets within the microfluidic channel. Should the volume of the droplet surpass that of the largest sphere that can fit within the channel, the droplet undergoes deformation into a disk, ellipsoid, or rod. (g–i) Optical microscope photographs captured for polyTPGDA particles with microsphere and rod-shaped morphology (g, i) and bismuth alloy ellipsoids generated through thermal solidification (h). (j) Schematic of the MF device for tunable-elasticity agarose microgels. The device measured 150 μm tall. The horizontal channel delivering mineral oil and serpentine channel at T-junction were 150 and 20 μm wide, respectively. The mixing channel was 250 mm long. (k) Optical microscope photographs of agarose droplets generated at a T-junction of the microfluidic device. Reproduced with permission from refs (232) and (233). Copyright 2005 Wiley-VCH and 2010 Elsevier, respectively.

The microbead fabrication procedure involves preparing a polymer solution loaded with a cell suspension. This is followed by the creation of droplets and their subsequent solidification. Droplet formation can occur through emulsification,^229^ extrusion,^230^ or microfluidics. Both emulsification and extrusion-based processes are conventional and have limitations in maintaining cell viability and consistency in maintaining the size and shape of microbeads. To overcome these drawbacks, microfluidics-assisted microbead generation was introduced. Using microchannels, an aqueous phase containing gelling agents and cells is broken into microdroplets by the sheer force generated by the second immiscible liquid called a continuous phase flowing in another channel. As shown in Figure 12, flow focusing, and T-junctions are the microfluidic designs studied extensively.

Flow-focusing microfluidic design (FFMD) generates microbeads irrespective of the composition, which is based on forcing two immiscible liquids through the orifice. The stream comprises the cells, the gelling agent is in the center, and the oil dispersion passes through two sides of the device. The oil phase surrounds the center phase, exerting pressure to break it into droplets at the orifice. The gelling of beads happens either through UV cross-linking or lowering the temperature. Additionally, microbead formation by enzymatic cross-linking was studied via this microfluidic design.^231^ In addition, by modifying the dimensions of the outlet channel, the microbead shape and size can be adjusted. Leijten and colleagues used various synthetic and natural microbeads to construct cell-laden microbeads.^231^ They developed biomaterial-based microbeads using FFMD to treat osteoarthritis in a rat model. Xu et al. explored the factors affecting the size and shape of microbeads produced via flow-focused microfluidics.^232^

In the design of a T-junction microfluidic system, the hydrogel precursor phase and immiscible phase flow in a direction perpendicular to each other. The procedure for producing agarose beads to enclose embryonic stem cells from mice was initially described^233^ The design involves guiding the cells containing the gelling agent via a microchannel, where they come into contact with a nonaqueous phase, often mineral oil, resulting in the formation of microspheres. Subsequently, exposing these microspheres to lower temperatures, specifically 2 °C, promotes the creation of microbeads. The utilization of the microfiber cell encapsulation technique offers significant benefits over microbeads in terms of simplified manufacturing and handling processes. Microfibers can be fabricated by electrospinning, microfluidics, or 3D printing techniques. The polymers most often used for microfiber production include alginate, gelatin, chitosan, PEG-4Mal, and PLGA.^234−237^ Alginate-based hydrogels have been investigated for various purposes, including the cryopreservation of red blood cells, cell therapy, and bone regeneration. Alginate was recently oxidized with 0.4% fibrinogen to imitate the extracellular matrix. The resulting mixture was then injected into calcium phosphate cement. Within 12 weeks, 40% of the bone defects were successfully repaired.^238^

## 8 Skin Tissue Regeneration Strategies

Skin tissue engineering (TE) is employed for treating deep wounds, such as burns, traumas, or illnesses, in cases where the body’s natural tissue regeneration is insufficient. The objective is to reinstate functioning and thickness while averting immunological reactions and implant rejection. The field of regenerative medicine quickly shifted its attention to tissue regeneration procedures, which hold promise for individuals afflicted with a range of injuries and disorders. These novel methods seek to utilize the body’s inherent regenerative abilities to restore injured skin tissue, leading to revolutionary progress in healthcare. Researchers are investigating various methods, such as stem cell treatment and tissue engineering, to determine how chronic wounds are treated.

### 8.1 Stem-Cell-Based Therapy

Stem cell-based therapy in wound care aims to increase the quality of wound healing, expedite the healing process, inhibit scar formation, and restore both skin and appendages. Stem cells can undergo self-renewal, asymmetric replication, and differentiation into several cell types, which renders them a very promising therapeutic option for chronic wounds.^241,242^ They regenerate depleted cells throughout the lifespan of an organism and release pro-regenerative cytokines. ESCs, induced pluripotent stem cells, and adult stem cells can potentially be used as sources for wound healing and regeneration.^243^ Nevertheless, the difficulty is in identifying the most advantageous origin and technique for processing and administering.

### 8.2 Scaffold

Skin scaffolds are essential for facilitating the development of skin tissue by delivering important signals for cell survival and specialization. Hybrids and composite materials have been utilized to augment the load-bearing ability of scaffolds. For example, scaffolds made of PCL-based fibers have been electrospun with gelatin, collagen/chitosan blends, and collagen/PEG/chitosan (CPCP).^244−246^ These materials have exhibited enhanced porosity, hydrophilicity, cell infiltration, and antibacterial efficacy. Growth factors have been incorporated into scaffolds to enhance the processes of granulation, regeneration, and promotion of cutaneous vascularization.^246^ The effectiveness of 3D-printed gelatin scaffolds, which are covered with sulfonated silk fibroin and basic fibroblast growth factor 2, has been demonstrated.^242^

### 8.3 Gene Therapy

Gene therapy utilizes genetic material to address or avert illnesses, possibly affecting hereditary conditions, cancer, and viral infections. Approaches include gene replacement, gene inactivation, and gene introduction. Gene therapy, while promising, is hindered by significant hazards related to its safety and efficacy. To address the issue of delayed cell development in mesenchymal stem cell (MSC) treatment, scientists have modified MSCs by introducing the *Cxcr6* gene. This gene allows the cells to produce a surface receptor called CXCR6, which can bind to a specific molecule called CXCL16. This molecule is found at high levels in wounds. The use of genetically engineered CXCL16 MSCs in treating diabetic wounds resulted in increased transformation of MSCs into keratinocytes and endothelial cells.^247^ Furthermore, the expression of the scavenger receptor gene is artificially increased in macrophages, leading to highly effective re-epithelialization and wound healing when these macrophages are cocultured with skin cells.^248^ Currently, clinical studies are being conducted for the gene treatment of recessive dystrophic epidermolysis bullosa (RDEB), a genodermatosis. This involves reactivating the mutant gene *COL7A1* via the herpes virus. The aims of reducing the wound area, duration, and time of wound closure were achieved with the use of *COL7A1* gene therapy.^249^

## 9 Stimuli-Responsive Ionic Gels in Tissue Regeneration

Tissue regeneration is a fascinating field that explores the body’s ability to repair and replace damaged or lost tissues. Among the promising materials identified in tissue regeneration, ionic gels are the most explored owing to their electrical charge. They have distinct physical and chemical characteristics and conductivities because of the presence of ionic moieties in their structures.^250^ Von Recum et al. conducted studies to examine how PNIPAAm scaffolds affect the enzymatic integrity of donor retinal cells.^251^ The temperature-sensitive material had no negative effect on the retinoid enzymatic profile. Furthermore, this material is one of the least harmful methods for cell development and detachment. Studies involving PNIPAAm-based coatings have shown that thermal gelling materials are necessary for ocular rehabilitation because they facilitate the removal of substrates during retinal cell regeneration.^252^ QPQGLAK peptides were added to poly(*N*-isopropylacrylamide-*co*-acrylic acid) hydrogels by Kim and Healy so that they may be used as an artificial extracellular matrix in the tissue regeneration process.^253^ The QPQGLAK sequence has been designated for particular degradation by osteoblast-produced matrix metalloproteinases (MMPs), such as MMP-13. The peptides were directly added to the hydrogels during the polymerization process. Initially, the sequence underwent functionalization by reacting with acryloyl chloride to produce acrylic end groups. The resulting product was then utilized as a monomer in a conventional chain polymerization reaction alongside *N*-isopropylacrylamide and acrylic acid. The outcome was a potential artificial extracellular matrix (ECM) that broke down in the body in response to a biological signal present in remodelled bone rather than randomly, as the PLA, PGA, or PLGA hydrogels do. This enables the hydrogel to function as a scaffold for bone regeneration that will not linger in the body and obstruct bone healing but will remain long enough for osteoblasts to migrate into it and initiate bone reformation.

pH- and redox-based ionic gels have been explored because of their ability to integrate easily and respond to tissue/organ or cellular activities. To this end, acylhydrazone linkages were combined with the Diels–Alder (DA) reaction to create double-cross-linked hyaluronic acid (HA)-based hydrogels.^254^ The DA cross-linking improved the structural integrity and mechanical strength of the network, whereas the acylhydrazone contributed to the pH-responsive behavior and self-healing properties. Simultaneously, the aldehyde groups that were added underwent a reaction with amines present in the nearby cartilage tissue. This reaction facilitated the effective integration of the hydrogel with the surrounding tissue. Similarly, the gels exhibited the ability to transition back and forth between a gel-like state and a liquid-like state within 20 min when the pH was adjusted to approximately 4. Compared with control DA cross-linked hydrogels, these materials exhibited enhanced adhesive capabilities, with an adhesive stress of 10 kPa compared with 1.5 kPa.

**Figure 13:**
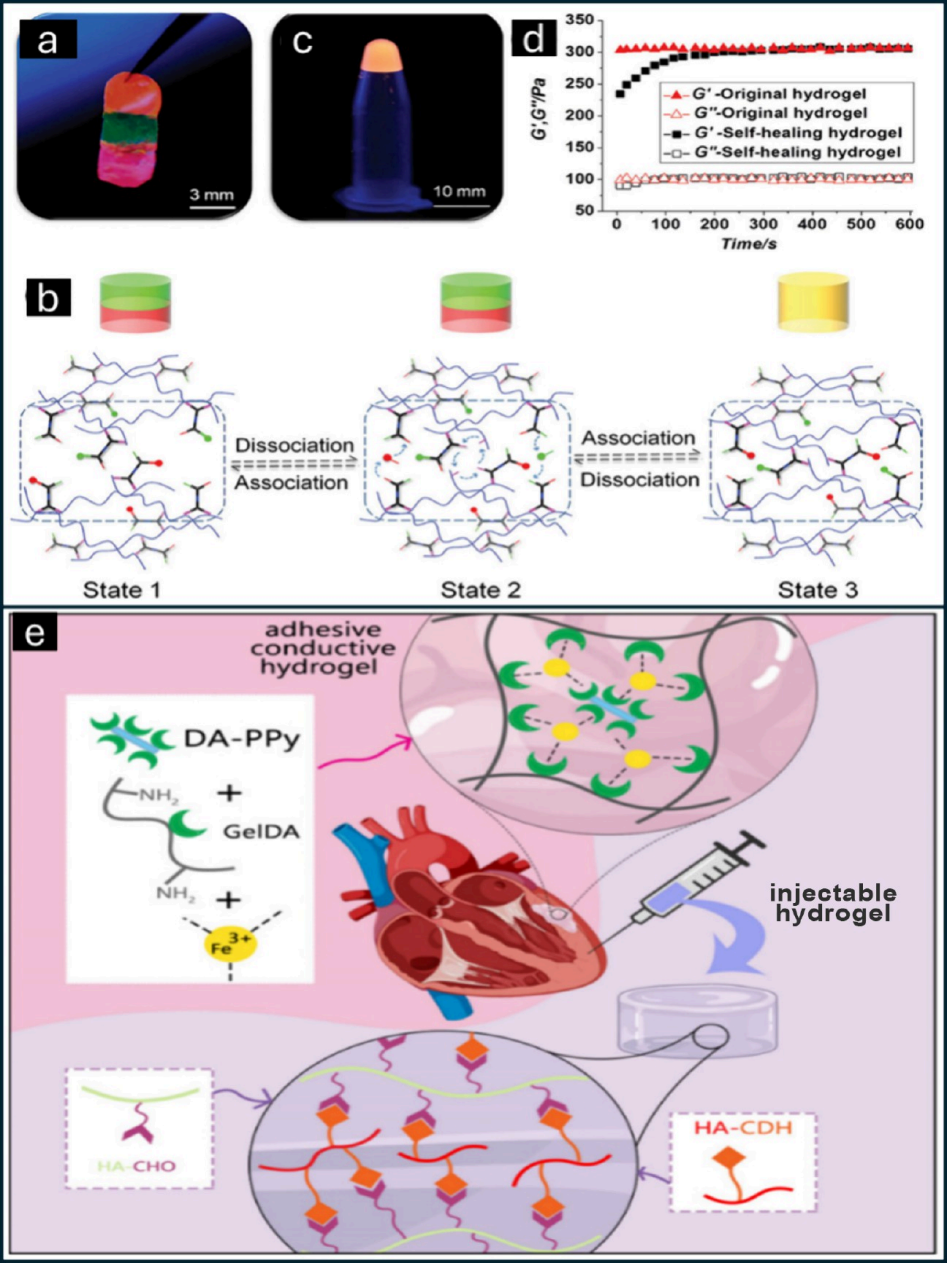
(a) A polypeptide–DNA hydrogel with self-healing properties was able to adhere from bottom to top, and the hydrogels consisted of 5(6)-ROX-modified, 5(6)-FAM-modified, and 5(6)-FAM/5(6)-ROX-modified polypeptide–DNA hydrogels. (b–d) The process by which self-healing occurs through the exchange of DNA chains (d), dye-treated hydrogels (c), and the mechanical properties of a polypeptide-DNA hydrogel (d). (e) The impact of using a combined therapy involving an adhesive hydrogel patch and an injectable hydrogel for the treatment of myocardial infarction. Adapted from ref (259). CC BY 4.0.

Protein–DNA complexes, HA, PEG, ELP, chondroitin sulfate, and silk fibroin are widely used to make self-healing hydrogels.^255^ Polypeptide, polyacrylamide, and carbon nanotube backbone polymers can generate novel hydrogels via self-healing linker DNA sequences. Temperature, UV, enzyme, pH, and light responsiveness make “X”-shaped DNA a good cross-linker. Li et al. synthesized polypeptide-DNA as a cross-linker (Figure 13a).^256^ Chain exchange events in the DNA double helix caused self-healing with different fluorophores (Figure 13b). DNA chain diffusion removes portions of the interfaces. The three colorful hydrogels appeared entirely and were adjusted to the container shape at 4 °C (Figure 13c). Figure 13d shows that the mechanical strength of the combined material reached 80% of its original value and that the hydrogel (4 wt %) that was cut into pieces could be repaired and recovered in 5 min. Nanomaterial-based hydrogels have been shown to improve thermal and electrical conductivity.^257^ Compared with virgin GelMA, the photo-cross-linkable gelatin methacryloyl (GelMA) hydrogel with carbon nanotubes (CNTs) has a constant beating rate and three times more homogeneous F-actin fibers. Shin et al. reported that the CNT-GeIMA hydrogel integrates and improves cell-to-cell communication in newborn rat cardiomyocytes. In addition, the reversible Schiff base reaction between aldehyde-oxidized alginate and amine gelatin groups controls H_2_S gas release to address complex myocardial infarction symptoms, such as increased vascularization and mechanical performance, adapt to dynamic cardiovascular conditions and is tightly integrated with myocardial tissue. In a different investigation by Wu et al., a conductive cardiac patch with injectable self-healing hydrogels was reported.^258^ These hydrogels were composed of a combination of gelatin-dopamine (GelDA) and dopamine-modified polypyrrole (DA-PPy) that exhibited ionic coordination. The Schiff base reaction between oxidized sodium hyaluronic acid (HA-CHO) and hydrazide hyaluronic acid (HHA) resulted in increased mechanical support. This hydrogel showed improved adhesion properties and induced angiogenesis in myocardial infarction. The combination of internal (hydrogel injection) and external (patch) therapy improved the storage modulus, conductivity, and gelation time of the hydrogel. This combination had a marked effect on enhancing cardiac function after the occurrence of myocardial infarction, surpassing the effects of single-hydrogel systems (Figure 13e).

Chitosan by itself does not exhibit temperature sensitivity as a polymer. However, when combined with β-glycerophosphate (β-GP), it produces a uniform gel structure when heated. β-GP functions as a catalyst in the sol–gel transition of chitosan under physiological pH and temperature conditions. The chitosan gelling system facilitates osteoblast proliferation and differentiation, making it a good option for bone tissue creation.^260^ Similarly, when synthetic polymers such as pNIPAAM were used, vascular endothelial cells experienced increased growth and demonstrated the formation of new blood vessels.^261^ Recent research employing peptide-based hydrogels demonstrated effective replication of the natural extracellular matrix. Elastin-like polypeptides (ELPs) and poly-D aspartic acid combine to create a temperature-sensitive firm structure that preserves the growth factors necessary for 3-D angiogenesis.^262^ Van Tomme and colleagues combined solutions of dextran ionic gel microspheres with opposing charges, integrating the injectability of ionic gel microspheres with physical cross-linking via ionic interactions.^263^ The gel network underwent degradation when exposed to low pH and high ionic strength. The ionic connections between cationic and anionic microspheres, which form a physical network, can be disrupted under stress. The gel reconstitutes upon the removal of tension, demonstrating the system’s reversible nature. Overall, it was hypothesized the utilization of this ionic gel in the fields of drug delivery and tissue engineering, to study the release of proteins or peptides.

Light-responsive hydrogels are attractive for biological applications when visible or UV light is used as a stimulant. A polymeric network and photoreceptive moiety make up their structure. Light stimuli change their physicochemical properties. Photochromic molecules receive optical signals and convert them into chemical signals. The functional hydrogel component receives the latter signal and initiates cross-linking to form a hydrogel. Acrylate-modified materials tend to undergo gelation when exposed to direct light or UV irradiation. Monteiro and colleagues conducted a comparative analysis between direct light and UV light while preparing methacryloyl hydrogels. Researchers have determined that UV light has the potential to generate hydrogels with microporous structures. Additionally, both light sources resulted in a high percentage of cell survival.^264^ Nevertheless, prolonged exposure to UV light might have detrimental effects on macromolecules. This feature motivated the investigation of visible light photoinitiators such as triethanolamine and eosin Y^265^ as well as hydrogel production methods that rely on visible light, such as stereolithography.^266^ Wang et al. reported the synthesis of short peptide conjugates containing merocyanine (MC), which were used to create hydrogels that exhibited several responsive properties.^267^ This hydrogel exhibited responsiveness to visible light irradiation, pH fluctuations, and the presence of Ca^2+^ ions, making it highly promising as photomemorable materials and in the field of tissue engineering.

Electrically responsive hydrogels receive electrical signals and induce the encapsulated cells to align and move inside the gel. The choice of polymer for constructing this hydrogel is critical; it must have excellent conductivity and promote cell proliferation. When combined with nonconducting polymers, graphene oxide may be utilized to create an electroconductive hydrogel.^268,269^ Smooth muscle cells and neuronal cells have been explored mostly in electrical stimulus studies. One study reported that the neuronal cell proliferation rate is proportional to the applied current amplitude.^268^ Smooth muscle cells exhibit a positive response to tissue development or collagen synthesis after a short stimulus, unlike neuronal cell types.^270^

Magnetic hydrogels can transfer substances throughout the body and create a gel at the desired location. Additionally, this capability enables the penetration of deeper tissues when subjected to an external magnetic field. Hydrogelation in deeper tissues occurs specifically with an alternating magnetic field.^271^ Typically, these hydrogels consist of a matrix that forms a hydrogel with magnetic nanoparticles. Magnetite nanoparticles and superparamagnetic iron oxide nanoparticles are commonly utilized because of their biocompatibility and lack of cytotoxicity. The incorporation of nanoparticles into a hydrogel matrix improves its storage modulus and mechanical characteristics.^272^

The biochemical/biological-responsive hydrogel consists of functional groups that are activated when they come into contact with the biological environment. For example, researchers have used polyethylene glycol hydrogels combined with matrix metalloproteinase enzyme-degrading peptides to investigate how applying cyclic stress during gel cleaving affects the development of stem cells. A study revealed that subjecting more quickly deteriorating gels to cyclic strain resulted in a significant 84% increase in stem cell proliferation, as well as an increase in collagen III and tenascin-C synthesis.^273^ Barthes and colleagues studied biological stimuli by combining fibroblasts with macrophages, monocytes, or interleukin-4 in a coculture. Coculturing was shown to provide a more stimulating microenvironment. Il-4 has a stimulatory effect on fibroblasts, leading to their increased proliferation. Macrophages increase the output of cytokines. In general, coculture conditions result in the formation of thick structures resembling tissues, which can be used as implantable systems.^274^

Taken together, multiple studies have aimed to develop highly stimuli-responsive ionic hydrogels for precise regulation of the delivery of therapeutics. However, to achieve precision, other factors must be considered, such as the immune response, response time, rates of degradation, surface hybridization, and inflammatory reactions. While recent studies on *in vivo* mouse-induced wound models have shown that ionogels can be biocompatible and effective in therapy when engineered correctly, the absence of safety data and uncertainty about their long-term impact remain major challenges for clinical application and regulatory approval. Furthermore, the chemical and biological properties of ILs make them very suitable for various applications, such as noninvasive techniques, innovative systems that respond to stimuli, delivery of biopharmaceuticals, and enhancement of medication pharmacokinetic profiles. Given the ongoing advancements in this sector and the development of new and enhanced ionogels for pharmaceutical purposes, conducting a thorough and multifaceted safety assessment of ILs and ionogels to determine their suitability for use in human applications is imperative. Nevertheless, the applications of self-healing ionic hydrogels have expanded due to the growing demand for accurate replication of the structures involved in cell formation and proliferation, as well as sustained drug delivery.

## 10 Conclusion and Prospects

A new class of materials called stimuli-based ionic gels combines the chemical flexibility of ILs and stimuli with the beneficial mechanical characteristics of biopolymers, such as their elasticity, stretchability, and integrity, with the ability to mend themselves. These materials have shown remarkable properties, including thermal and electrochemical stability, strong ionic conductivity, and enhanced biocompatibility while exhibiting minimal cytotoxicity and sensitivity to environmental cues. These have promising futures in cutting-edge therapeutic delivery systems, among other domains. A wide range of therapeutic agents, including cells, nanoparticles, proteins, peptides, genes, and conventional molecules, might be encapsulated in these gels, opening numerous possibilities for their use in pharmaceutical research. However, low biocompatibility and high toxicity are common problems with these ILs, especially when they are used as cross-linkers. Although there are alternatives to cross-linkers, there is an urgent need in the biomedical industry for more biocompatible ILs. Examples of such ILs include those based on cholinium cations and glycine-betaine analogues.^147,282,283^

Stimuli-responsive polymer-based ionic gels share similar properties with biological tissues, making them very suitable for clinical treatment purposes. Research in this field is well established, yet there remains a significant disparity compared with their clinical application. Therapeutic applications are facilitated by advancements in biocompatibility, biodegradability, stability, and simplicity. Transitioning stimuli-responsive ionic gels to clinical applications in the future will pose significant challenges. Stimuli-responsive ionic gels can perceive and react to stimuli, as discussed earlier. However, the majority of these gels are limited in providing basic feedback to a single stimulus, which fails to fulfill the demands of emulating real systems with complex abilities. Designing ionic polymers that can react to many, or different stimuli is a viable method to solve the aforementioned issues. In the future, the next phase in the development of stimuli-responsive polymer-based ionic gels may involve synthesizing a polymer that can respond to many stimuli and finding ways to control the combined effects of multiple responsive polymers.

ILs have significant potential for advancing the development of smart hydrogels that respond to stimuli, as discussed earlier. Currently, there is ongoing research in the frontier area of IL-based hydrogels that incorporate magnetic and chemical processes. However, owing to the lack of significant studies in these fields, there is a need for constant efforts to create other types of responsive hydrogels that can also be applied to chemical-responsive hydrogels. This can be achieved by utilizing various applications of ILs to create a comprehensive range of smart and responsive hydrogel polymers.

In the context of wound healing, solutions for treating complex chronic wounds, namely, non-self-healing diabetic ulcers, are lacking. However, the latest advancements in the synthesis of multifunctional stimuli-responsive polysaccharide-based gels in wound dressings have provided optimizm for effectively treating both acute and chronic wound conditions. These gels have advantages over traditional wound dressing methods. They can be activated at the site of the wound and provide a specific response to the affected area. This promotes wound healing and skin reconstruction while also providing antimicrobial protection during the healing process. Stimuli-responsive polysaccharide gels can combine various properties and responses, depending on the specific application area, wound type, and conditions. While recent studies on live mouse models have generally shown that ionic gels can be biocompatible and therapeutically effective if designed correctly, the absence of safety data and uncertainty about their long-term impact on human health remain major challenges for clinical use and regulatory approval. Notably, cell culture-based assessment of toxicity studies should also be performed at the initial stages to maintain the effectiveness of these stimuli-responsive ionic gels, which are still lacking.

The consistent incorporation of new chemistries into stimuli-responsive ionic hydrogels that can mimic native tissue architecture and provide controlled cell and signaling cue distributions will lead to significant advancements in the development of supramolecular hydrogels in the future. This should be a continuous process to create supramolecular gels that may regulate the spatiotemporal release of biologics. However, designing and developing these gels might be challenging, as they must replicate the structural, mechanical, and dynamic properties of natural tissue. By responding and adapting to mechanical and biological changes in the scaffold, “smart stimuli-based iongels” can be developed that may regulate the controlled release of biologics or therapeutics in beneficial spatiotemporal patterns across predetermined time points. One of the main goals of many tissue engineering structures is cell infiltration and, by extension, tissue integration, which relies on material chemistry as well as cell adhesion. To meet the requirements of stiffer scaffolds, a slower rate of erosion and clearance, recurrent self-healing, or a combination of these factors, enhancing the mechanical properties of these ionic gels may be necessary. This can be accomplished through the emergence of biomaterials that possess the ability to self-heal multiple times under physiological conditions. Taken together, stimuli-responsive self-healing ionic gels are gaining popularity as possible solutions, particularly in the areas of wound healing management, tissue engineering and drug delivery.
